# The predominance of nucleotidyl activation in bacterial phosphonate biosynthesis

**DOI:** 10.1038/s41467-019-11627-6

**Published:** 2019-08-16

**Authors:** Kyle Rice, Kissa Batul, Jacqueline Whiteside, Jayne Kelso, Monica Papinski, Edward Schmidt, Alena Pratasouskaya, Dacheng Wang, Rebecca Sullivan, Christopher Bartlett, Joel T. Weadge, Marc W. Van der Kamp, Gabriel Moreno-Hagelsieb, Michael D. Suits, Geoff P. Horsman

**Affiliations:** 1 Department of Chemistry & Biochemistry, Wilfrid Laurier University, Waterloo, ON N2L 3C5 Canada; 2 Department of Biology, Wilfrid Laurier University, Waterloo, ON N2L 3C5 Canada; 3 School of Biochemistry, University of Bristol, Bristol, BS8 1TD UK

**Keywords:** Transferases, Biosynthesis, X-ray crystallography

## Abstract

Phosphonates are rare and unusually bioactive natural products. However, most bacterial phosphonate biosynthetic capacity is dedicated to tailoring cell surfaces with molecules like 2-aminoethylphosphonate (AEP). Although phosphoenolpyruvate mutase (Ppm)-catalyzed installation of C-P bonds is known, subsequent phosphonyl tailoring (Pnt) pathway steps remain enigmatic. Here we identify nucleotidyltransferases in over two-thirds of phosphonate biosynthetic gene clusters, including direct fusions to ~60% of Ppm enzymes. We characterize two putative phosphonyl tailoring cytidylyltransferases (PntCs) that prefer AEP over phosphocholine (P-Cho) – a similar substrate used by the related enzyme LicC, which is a virulence factor in *Streptococcus pneumoniae*. PntC structural analyses reveal steric discrimination against phosphocholine. These findings highlight nucleotidyl activation as a predominant chemical logic in phosphonate biosynthesis and set the stage for probing diverse phosphonyl tailoring pathways.

## Abstract (web summary)

Phosphonate modifications can be present on microbial cell surfaces. Here the authors perform bioinformatics analyses and observe a widespread occurrence of nucleotidyltransferase-encoding genes in bacterial phosphonate biosynthesis and functionally characterize two of the identified phosphonate specific cytidylyltransferases (PntCs) and determine the crystal structure of *T. denticola* PntC.

## Introduction

**Fig. 1:**
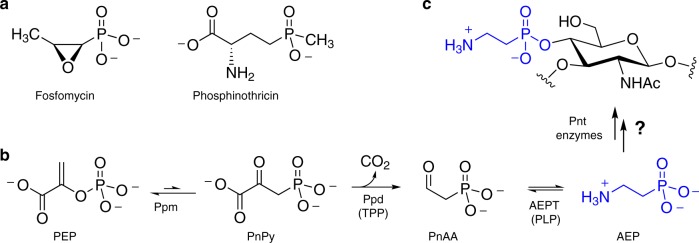
Phosphonates in nature. **a** Small molecule bioactive natural products fosfomycin and phosphinothricin. **b** Biosynthesis of the common phosphonate AEP from phosphoenolpyruvate (PEP) via the intermediacy of phosphonopyruvate (PnPy) and phosphonoacetaldehyde (PnAA). Cofactors in parentheses are thiamine pyrophosphate (TPP) and pyridoxal 5′-phosphate (PLP). Unknown phosphonyl tailoring (Pnt) biosynthetic steps lead from AEP to **c**, cell surface phosphonoglycans like that isolated from *Bacteroides fragilis*

With stability and bioactivity imparted by a characteristic carbon–phosphorus bond, phosphonates are unusual and relatively unexplored biological molecules. Since the discovery of 2-aminoethylphosphonate (AEP) in nature six decades ago^1^, phosphonates and phosphinates have gained recognition as a small but commercially successful class of natural products exemplified by the antibiotic fosfomycin and the herbicide phosphinothricin (Fig. 1a)^2,3^. Although exploration of these molecules and their biosynthesis is still in its infancy, bioinformatic analyses have revealed widespread genetic capacity to produce chemically diverse phosphonates^4–6^. For example, ~5% of sampled microbes encode phosphoenolpyruvate mutase (Ppm)^4^, which catalyzes rearrangement of phosphoenolpyruvate (PEP) to the C–P bond-containing product phosphonopyruvate (PnPy) (Fig. 1b)^7^. Despite this predicted ubiquity, natural product classes like polyketides and nonribosomal peptides have been far more intensively investigated, and the relative dearth of characterized phosphonates partly reflects technical challenges associated with high polarity and lack of chromophores^8^. However, the few phosphonate biosynthetic gene clusters that have been characterized have revealed unusual enzymes catalyzing unprecedented chemical transformations^9^. These findings highlight the gap between current biosynthetic knowledge and future discovery potential, and motivate continued investigation of biological phosphonates.

Even more overlooked than freely diffusible small molecule phosphonates are those that modify, or tailor, cell surfaces. Comprehensive bioinformatic analysis of microbial *ppm* gene distribution predicted that most putative phosphonate biosynthetic gene clusters encode cell wall phosphonoglycans and phosphonolipids^4^. Although a handful of phosphonylated glycans and lipids have been structurally identified—such as the AEP-modified capsular polysaccharide from *Bacteroides fragilis*^10^ (Fig. 1c)—almost nothing is known about their biosynthesis or biological roles. Modulating biological function by adding or removing small molecules on cell surface materials has long been recognized as a key mechanism of bacterial adaptation and virulence. Common cell surface modifications include the addition of acetyl^11^, pyruvyl^12^, phosphoethanolamine^13,14^, and phosphocholine (P-Cho)^15–17^ groups to lipids and glycans. Indeed, P-Cho is a virulence factor encoded by the Lic pathway in pathogens, such as *Streptococcus pneumoniae*^15,18^, and *lic* gene disruption impedes P-Cho decoration of teichoic acids and attenuates *S. pneumoniae* virulence^19–21^. Understanding the biosynthetic logic of these modifications therefore represents an important goal with therapeutic implications, as phosphonates may similarly modulate virulence.

To address the gap between bioinformatic prediction and biosynthetic knowledge, we are characterizing several biosynthetic gene clusters predicted to encode cell wall phosphonoglycans or phosphonolipids. For instance, guided by the presence of *ppm* genes, we previously identified gene expression and/or phosphonate production from anaerobic bacteria *Atopobium rimae*, *Treponema denticola*, and *Olsenella uli*^22^, each of which have been associated with disease of the human oral cavity^23–25^. Commonly associated with *ppm* and found in these gene clusters are *ppd* and *aept*, which respectively, encode PnPy decarboxylase (Ppd) and AEP transaminase (AEPT) to generate AEP (Fig. 1b)^26,27^. Unfortunately, additional genes commonly found in predicted phosphonyl tailoring gene clusters have not been described, and the biosynthetic steps leading to phosphonyl modifications remain unknown (Fig. 1). Herein we reveal widespread occurrence of nucleotidyltransferase-encoding genes in phosphonate biosynthesis and characterize two representative enzymes as phosphonate-specific cytidylyltransferases from the Gram-positive actinobacterium *A. rimae* and the Gram-negative spirochete *T. denticola*. These enzymes efficiently activate AEP, presumably for subsequent capture by carbohydrate or lipid nucleophiles. We propose the phosphonyl tailoring (*pnt*) nomenclature for these gene clusters, and specifically use PntC for AEP cytidylyltransferase based on its sequence and biosynthetic similarity to LicC of the P-Cho tailoring (Lic) pathway. Indeed, in contrast to PntC, LicC from *S. pneumoniae* (Spn-LicC) strongly prefers P-Cho over AEP as a substrate for cytidylyl activation. Structure determination of PntC from *T. denticola* (Tde-PntC) and molecular dynamics analyses indicate that PntC selectivity for the smaller AEP substrate is primarily driven by active site steric constraints. Overall, identifying this remarkably widespread family of phosphonate-specific PntC cytidylyltransferases sets the stage for deciphering the biosynthetic roles of CMP-phosphonate conjugates and the biological consequences of this underexplored cell surface chemistry.

## Results

### Nucleotidyltransferases are enriched in phosphonate biosynthesis

**Fig. 2:**
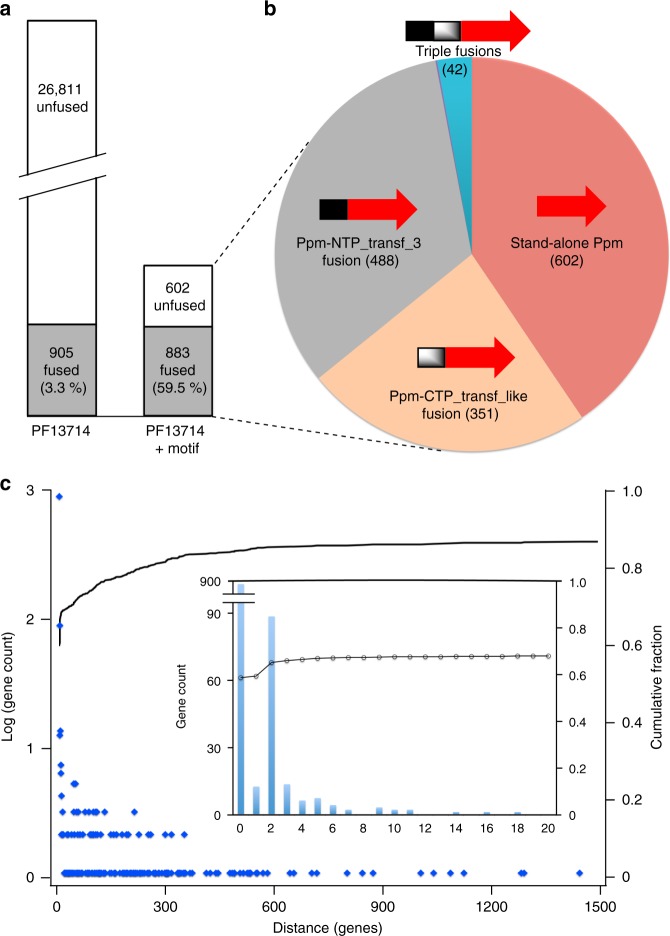
Genomic relationships between *ppm* and nucleotidyltransferase-encoding genes. **a** Left: 27,716 non-redundant Ppm protein sequences were identified in NCBI’s refSeq prokaryotic genome database that matched the PF13714 HMM, of which 905 were fused to other domains. Right: After filtering for the presence of the characteristic Ppm motif (EDKXXXXXNS), 26,231 presumed non-Ppm sequences were removed. Only 22 of these removed sequences were fused to other domains. **b** Summary of the remaining 1485 predicted Ppm proteins and inventory of fusion status: Ppm alone (red, 40.5%); PF12804, MobA-like NTP transferase (NTP_transf_3; gray, 32.9%); PF01467, Cytidylyltransferase-like (CTP_transf_like; orange, 23.6%); not visible are two genes matching PF02775 and PF02776 (both TPP-binding domains), which corresponds to 0.1% of non-redundant Ppm proteins. The blue slice represents 42 triple fusion proteins (2.8% of all Ppm proteins) with Ppm fused to both PF12804 and PF01467. **c** Distribution of genomic distances (in genes) between *ppm* and the nearest of either PF12804 or PF01467. Blue dots represent logarithm of the number of genes counted for each distance (corresponding to left y-axis), and the line represents the cumulative fraction of genomic distances (right *y*-axis); 59.3% of nearest nucleotidyltransferase genes (those closest to *ppm*) are fused to *ppm* (distance = 0), and only single counts occur at distances greater than ~350 genes. *Inset*: Number of genes for each distance up to 20, illustrating that two-thirds of nucleotidyltransferase genes are within ~5 genes of *ppm*

To identify enzymes commonly associated with Ppm, we assessed Ppm protein sequences for the presence of fused domains. Precedent for active Ppm fusion proteins was recently established in the *Streptomyces wedmorensis* fosfomycin biosynthetic pathway. The Ppm enzyme Fom1 possesses an *N*-terminally fused cytidylyltransferase (CyTase) domain catalyzing cytidylyltransfer from CTP to hydroxyethylphosphonate (HEP) to generate CMP-HEP^28^. Inspired by this example of a biosynthetically relevant Ppm-fused domain, we generated a Ppm fusion inventory (Fig. 2). From the NCBI refSeq database containing 113,086 genomes we identified 146,399 proteins in 72,350 genomes matching the hidden Markov model (HMM) for the Ppm/isocitrate lyase Pfam family (PF13714). After filtering out identical sequences we were left with 27,716 non-redundant protein sequences matching PF13714, of which only 905 (3.3%) were fused to another Pfam domain (Fig. 2a). Of the 27,716 total sequences, only 1485 possessed the Ppm-specific EDKXXXXXNS motif^4^. Surprisingly, almost all fusions (883 out of 905) possessed this motif, indicating that the majority of Ppm enzymes (59.5%) are fused to other domains (Fig. 2a) in what represents an unusual and potentially distinguishing feature of the Ppm enzyme family. Indeed, only 40.5% of Ppm enzymes were ‘stand-alone’ proteins not fused to any other domain, and most Ppm enzymes were fused to the MobA-like NTP transferase domain (PF12804, 33.0%), to the cytidylyltransferase-like domain (PF01467, 23.6%), or both (2.9%, Fig. 2b). PF12804 is exemplified by Spn-LicC^29^, and PF01467 by the Fom1 CyTase^28^.

**Fig. 3:**
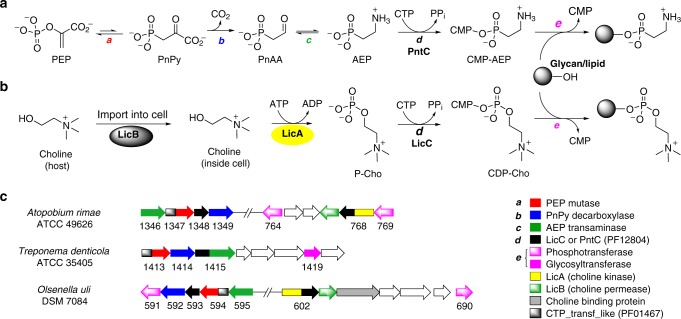
Bacterial cell surface modifications employing cytidylyl activation. **a** Proposed phosphonyl tailoring (Pnt) pathway logic based on its similarity to **b** the lipopolysaccharide core (Lic) pathway. **c** Putative gene clusters for three oral anaerobes under study, highlighting relevant genes for phosphocholine (P-Cho) and phosphonate tailoring. Black-colored genes are proposed to encode phosphonate-tailoring cytidyltransferases (PntCs) when located near *ppm* genes (red), and P-Cho cytidyltransferases (LicC) when located near other *lic* genes (e.g. those encoding LicA shown in yellow). For clarity, annotations for uncolored genes are not included

This predominance of nucleotidyltransferase domains fused to Ppm enzymes was unexpected and supports a co-evolutionary trajectory of the two catalytic activities. In bacteria, co-evolution of functionally related genes is generally observed as co-localization, or clustering, on the chromosome^30^. A compilation of calculated distances, in number of genes, between *ppm* and the nearest gene encoding either PF12804 or PF01467 clearly illustrated a tendency for these genes to be in close proximity (Fig. 2c). For instance, 59.3% of all nearest nucleotidyltransferases were fused to *ppm* (distance = 0 genes). The next most frequent distance was two genes away, and about two-thirds (67.7%) of nucleotidyltransferases were within five genes of *ppm* (Fig. 2c inset). Note that these calculations identify only the nearest nucleotidyltransferase in a given gene cluster and therefore do not account for gene clusters possessing more than one nucleotidyltransferase. For example, the gene clusters in Fig. 3c possess nucleotidyltransferases that are both *ppm*-fused as well as one or two genes away. In these cases only the nearest nucleotidyltransferase would be tabulated, which for all three would be the fused domain at a distance of zero. In summary, this proximity bias indicates a functional relationship between Ppm and nucleotidyltransferases, and strongly implies an important role for the latter in phosphonate biosynthesis.

Interestingly, many *ppm*-associated nucleotidyltransferases are annotated as LicC-like cytidylyltransferases, which provides an important clue regarding possible biosynthetic function. The *lic* (lipopolysaccharide core) operon catalyzes choline import, phosphorylation and attachment of P-Cho to a glycan substrate destined for the cell wall^18,31^. Mirroring the chemical logic of the eukaryotic Kennedy pathway, a LicC cytidylyltransferase catalyzes formation of CDP-choline from CTP and P-Cho (Fig. 3b). LicC from *S. pneumoniae* (Spn-LicC) has been structurally and biochemically characterized^29,32,33^, and gene disruption attenuated virulence in a mouse model^21^. Overall, the similarities between P-Cho and AEP and genomic proximity of *ppm* and *licC*-like genes suggested that phosphonates may be activated as CMP-conjugates in a manner analogous to P-Cho activation by LicC (Fig. 3a).

### PntC enzymes preferentially activate AEP

Intrigued by the genomic proximity of *ppm* and *licC*-like cytidylyltransferases, we sought to characterize the putative phosphonate-specific nucleotidyltransferases Oul593, Ari1348 and Tde1415. We observed transcription of *oul593* and *ari1348* in pure cultures (Supplementary Fig. 1), and previously reported chemical shifts consistent with phosphonates via solid-state ^31^P NMR analysis of *T. denticola* and *O. uli* whole cells^22^. Furthermore, each genomic neighborhood encodes AEP production and biosynthetic machinery associated with cell wall glycans, such as LicD-like phosphotransferases or glycosyltransferases (Fig. 3c). In fact, both *A. rimae* and *O. uli* possess *lic* genes relatively nearby on the chromosome. The Ppm protein sequences also phylogenetically cluster into phosphonoglycan/lipid clades^4,22^, which further suggests that these phosphonates are directed to the cell surface. To evaluate the substrate preference of putative phosphonate nucleotidyltransferases relative to Spn-LicC, we expressed and purified Ari1348, Tde1415, and Spn-LicC, but Oul593 was insoluble (Supplementary Fig. 2). Dynamic light scattering (DLS) analysis indicated monomeric quaternary structures for Spn-LicC and Ari1348 (Supplementary Fig. 3), as previously observed for Spn-LicC^33^. In contrast, Tde1415 has a predicted monomeric molecular weight of ~70 kDa with DLS data indicating a dimer of ~146 kDa.

**Fig. 4:**
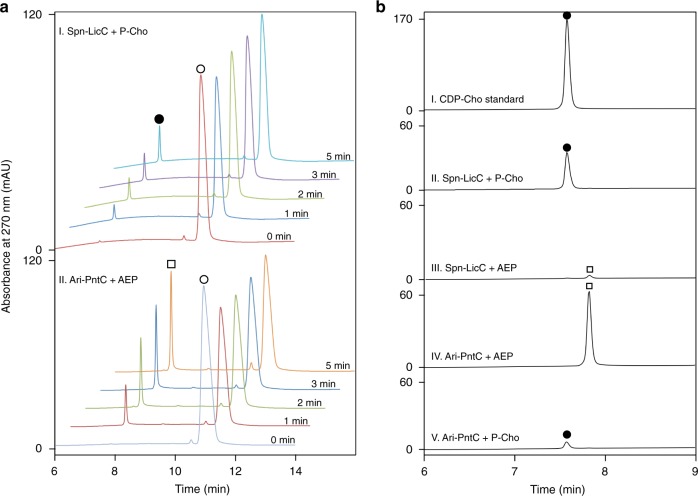
HPLC chromatograms of reactions catalyzed by Spn-LicC and Ari-PntC (Ari1348). **a** Time-resolved reaction of CTP (open circle) with: I, P-Cho and Spn-LicC to make CDP-Cho (filled circle), and II, AEP and Ari-PntC to generate CMP-AEP (open square). **b** Spn-LicC more readily produces CDP-Cho (panel II) than CMP-AEP (panel III); in contrast, Ari-PntC preferentially generates CMP-AEP (panel IV) compared to CDP-Cho (panel V) after 30 min of reaction time. All reactions contained 50 nM enzyme, 1 mM of either AEP or P-Cho, 4 mM CTP, and 7 mM MgCl_2_ in 50 mM Tris–Cl, pH 8.0

Cytidylyltransferase activity detected using an HPLC assay revealed time-dependent formation of CDP-choline (CDP-Cho) and CMP-AEP by Spn-LicC and Ari1348, respectively (Fig. 4a). CDP-Cho was verified by comparison to commercially sourced authentic standard and high-resolution mass spectrometry (expected mass, 488.1073; observed, 488.1071). CMP-AEP was identified based on high-resolution mass spectrometry and multinuclear NMR (Supplementary Figs. 4–7). Overall, these results clearly demonstrated Ari1348-catalyzed formation of CMP-AEP and imply shared biosynthetic logic for P-Cho and phosphonate tailoring pathways (Fig. 3).

Although activity was observed for each enzyme with its proposed substrate, a comparison of substrate preference further supported our cognate substrate assignments and prompted the adoption of new nomenclature to describe this class of phosphonate-specific enzyme. Previous work revealed a 2700-fold higher specificity constant for Spn-LicC towards P-Cho relative to phosphoethanolamine (Table 1), but activity towards phosphonate substrates was not examined^33^. Our HPLC assays revealed poor cross-reactivity with non-cognate substrates (Fig. 4b). Specifically, Ari1348 clearly preferred AEP over P-Cho under the same conditions, signifying phosphonate cytidylyltransferase activity. In addition, both Ari1348 and Tde1415 exhibited strong activity towards CTP, limited activity towards ATP, and no activity detected for GTP (Supplementary Fig. 8). Similar limited reactivity towards ATP was previously observed in the Fom1 CyTase domain^28^ and Spn-LicC^32,33^. Based on phosphonate preference and functional analogy to LicC, we propose phosphonyl tailoring cytidylyltransferase (PntC) terminology to distinguish these enzymes from P-Cho-specific LicC cytidylyltransferases (Fig. 3).

**Table 1:** Steady-state kinetic constants of cytidylyltransferases^a^

Enzyme	Substrate	*k*_cat_ (s^−1^)	*K*_M_ (mM)	*k*_cat_/*K*_M_ (M^−1^ s^−1^)	Specificity^b^	Ref.
Ari-PntC	AEP	3.7 ± 0.1	0.012 ± 0.001	3.2 × 10^5^	0.0023	This work
	ChoP^c^	0.7 ± 0.3	1.0 ± 0.8	7.2 × 10^2^		
Spn-LicC	AEP	0.72 ± 0.05	0.3 ± 0.1	2.3 × 10^3^	200	This work
	ChoP^d^	1.06 ± 0.04	2.4( ± 0.6)x10^−3^	4.5 × 10^5^		
	ChoP	3.6	0.060	6.0 × 10^4^	2700	^30^
	PEtn	0.031	1.43	22		
	ChoP	17.5	0.39	4.5 × 10^4^		^29^
	ChoP	37	0.066	5.6 × 10^5^		^26^
Tde-PntC	AEP	1.05 ± 0.05	0.016 ± 0.005	6.8 × 10^4^	ND	This work
	ChoP	ND^e^	ND	ND		

*PEtn* phosphoethanolamine

^a^Standard errors are included for data generated in this work

^b^Ratio of specificity constants (*k*_cat_/*K*_M_) for ChoP:AEP (or PEtn)

^c^Substrate inhibition observed with *K*_S_ = 4 ± 1 mM

^d^Substrate inhibition observed with *K*_S_ = 6.4 ± 0.9 mM

^e^ND, not determined due to low activity

The strict substrate specificity implied by the HPLC analyses of Fig. 4b prompted quantitative investigation via steady-state kinetic analysis. Using a coupled assay to detect pyrophosphate release^34,35^, our initial rate measurements of Spn-LicC towards P-Cho with saturating concentrations of CTP revealed a *k*_cat_ of 1.06 ± 0.04 s^−1^ and *K*_M_ of 2.36 ± 0.65 µM, affording a *k*_cat_/*K*_M_ comparable to previous reports^29,32,33^ (Table 1, Supplementary Fig. 9). In contrast, AEP as a substrate yielded *k*_cat_ and *K*_M_ values of 0.723 ± 0.054 s^−1^ and 318 ± 129 µM, respectively, revealing a 200-fold preference for P-Cho over AEP. Ari-PntC (Ari1348) exhibited the opposite preference, with a 440-fold higher specificity constant for AEP versus P-Cho (Table 1, Supplementary Fig. 9). Similarly, Tde-PntC (Tde1415) was not sufficiently active towards P-Cho to obtain steady-state kinetic data, but activity towards AEP was similar to Ari-PntC (Table 1). Overall, clear preference for AEP as a substrate supports assignment of these enzymes as phosphonate-specific cytidylyltransferases, or PntCs.

### Molecular determinants of specificity are primarily steric

Structural studies were undertaken to understand PntC substrate selectivity for AEP versus P-Cho. In contrast to Ari-PntC, the larger two-domain protein Tde1415 was readily crystallized to afford a 2.72 Å structure of the apo enzyme and a 1.95 Å structure of the product complex obtained in the presence of CTP and AEP substrates (Supplementary Table 1). As predicted from gene annotations (Fig. 3c), the overall structure of the enzyme included an *N*-terminal PntC connected to a *C*-terminal AEPT domain (Supplementary Fig. 10). The classic type I aminotransferase fold of the AEPT domain is very similar to AEPT from *Salmonella typhimurium*, which is the only other AEPT structure known^36^. Interestingly, neither of these structures possesses a covalently linked pyridoxal 5′-phosphate (PLP) cofactor, and the colorless Tde1415 solution was consistent with a missing internal aldimine chromophore. To our knowledge, these two enzymes and the alanine-glyoxylate aminotransferase from *Anabaena*^37^ are the only crystallographic examples of non-covalently bound PLP. Nonetheless, aminotransferase activity was detected as ^31^P NMR chemical shifts consistent with the transformation of AEP to phosphonoacetaldehyde (PnAA, Supplementary Fig. 11) and confirmed by mass spectrometry (Supplementary Fig. 12). The AEPT domain of Tde1415 possesses PLP and the requisite active site features to support classical aminotransferase catalysis (Supplementary Fig. 13).

**Fig. 5:**
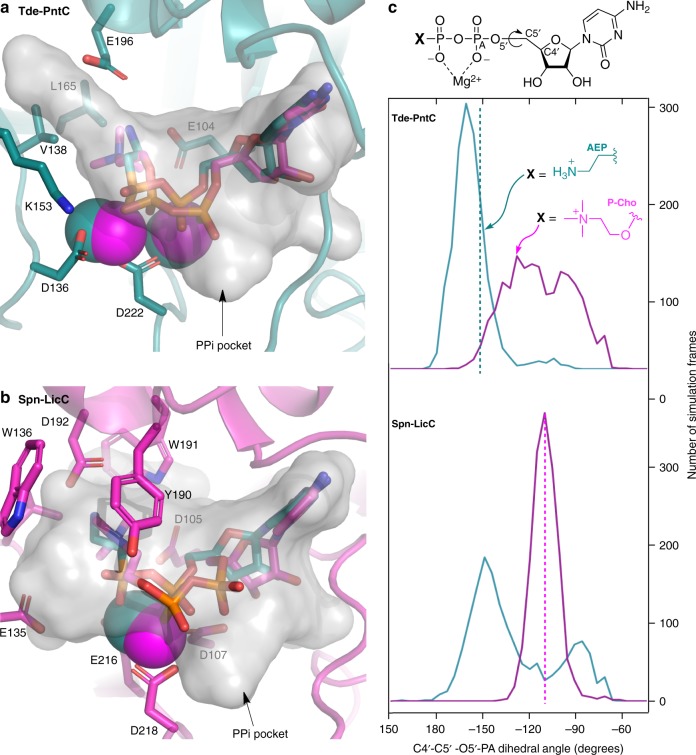
Molecular dynamics simulations of Tde-PntC (top) and Spn-LicC (bottom). **a** Representative conformations of the Tde-PntC active site with cognate ligand CMP-AEP and Mg^2+^ ions colored teal; non-cognate ligand CDP-Cho and Mg^2+^ ions are magenta. The transparent gray surface representation denotes the 287 Å^3^ cavity calculated from the crystal structure (minus CMP-AEP) using the Roll algorithm of POCASA^42^. **b** Representative conformations of the Spn-LicC active site showing the cognate ligand CDP-Cho and Mg^2+^ ion colored magenta; non-cognate ligand CMP-AEP and Mg^2+^ are colored teal. Transparent gray surface outlines the 381 Å^3^ cavity calculated from the 1JYL crystal structure coordinates after removing CDP-Cho (26). **c** Histogram of C4′-C5′-O5′-PA dihedral angles sampled during 10 × 150 ps independent simulations for each protein:ligand pair, using the same color scheme for each ligand (CMP-AEP = teal; CDP-Cho = magenta). Dotted lines represent crystallographically observed dihedral angles

The crystal structure of the PntC domain of Tde1415 in complex with the CMP-AEP product possesses a binding orientation reminiscent of the previously reported Spn-LicC:CDP-Cho structure (PDB 1JYL, Fig. 5)^29^. Strikingly, the presence of two adjacent magnesium ions in the Tde-PntC active site contrasts with the single ion observed in the Spn-LicC complex, and each apo-enzyme has one less metal ion. Consistent with our assignment of the metals as magnesium ions, the temperature factors increased from 31.3 to 62.7 Å^2^ when Zn^2+^ was included in crystallographic refinements. In addition, EDTA treatment inactivated Tde1415, but activity was restored by the addition of Mg^2+^ or Zn^2+^ but not Ca^2+^ (Supplementary Fig. 14). Varying metal ion content is well established among phosphate-processing enzymes. For example, a two-magnesium mechanism is favored for CMP-Kdo synthetase (KdsB)^38,39^, FomD employs two metals for cytidylyl (*S*)-2-hydroxypropoylphosphonate hydrolysis^40^, DNA polymerases commonly employ a two-metal ion mechanism with evidence for an additional third divalent metal ion^41^, and terpene synthases require three metal ions^42^. Overall, in contrast to Spn-LicC, which loses a salt bridge between Arg129 and a metal-coordinating residue Glu216, the metal coordination sphere of Tde-PntC does not undergo major changes upon ligand binding (Supplementary Fig. 10).

The crystal structures of Spn-LicC^29^ and Tde-PntC provide an opportunity to probe the selectivity determinants for respective cognate substrates P-Cho and AEP, which are present in the active sites as CMP-conjugate products. The two substrates clearly differ in size, and possibly in charge; the positive charge of the quaternary amine of P-Cho does not necessarily occur at the primary amine of AEP. Although primary amines are typically positively charged (p*K*_a_~10)^43^, active site p*K*_a_ perturbations could occur^44^ to neutralize AEP. However, few clues exist from the Tde-PntC crystal structure, which reveals water, Glu196, and Glu104 positioned to interact with the primary amine of AEP via hydrogen bonds. Intriguingly, the equivalent positions in Spn-LicC (D192 and D105) are occupied by smaller Asp side chains (Fig. 5), which may provide more room to accommodate the larger P-Cho substrate. In addition, Spn-LicC possesses a composite aromatic box for binding the choline quaternary amine via cation–π interactions (Fig. 5)^45^. Notably, Ari-PntC also possesses the equivalent aromatic residues, suggesting that charge may not be a key determinant of P-Cho versus AEP selectivity, and highlighting that Ari-PntC is more closely related to Spn-LicC than Tde-PntC (Supplementary Fig. 15). Therefore, protein cavity volumes were calculated^46^ to evaluate the effects of steric restriction against the larger P-Cho substrate in the Tde-PntC active site. The larger pocket volume of Spn-LicC (~381 Å^3^) compared to Tde-PntC (~287 Å^3^) (Fig. 5) corresponds to the difference of ~64 Å^3^ in molecular volume between CDP-Cho and CMP-AEP, or the ~75 Å^3^ volume of the choline trimethylamine moiety^47^.

To further probe the effects of steric constraint on substrate selectivity, we pursued molecular dynamics simulations. In addition to the enzyme:product crystal structures of Spn-LicC:CDP-Cho and Tde-PntC:CMP-AEP, we also modeled non-cognate enzyme:product pairs Spn-LicC:CMP-AEP and Tde-PntC:CDP-Cho using 10 × 150 ps independent trajectories (1.5 ns in total) for each complex (Fig. 5). Notably, the Tde-PntC active site did not readily accommodate the additional volume of CDP-Cho, and significant conformational changes in the ligand occurred immediately during the simulations. Specifically, the choline moiety was forced towards cytidine, with a concomitant shift in the phosphate backbone enabled by rotation about the ribose C5′–O5′ bond. The distribution of this dihedral angle during the Tde-PntC simulations indicates: (i) the magnitude of this shift for the non-cognate ligand CDP-Cho, and (ii) the comparatively narrow distribution of dihedral angles accessed by the cognate ligand CMP-AEP (Fig. 5c). Similar results were obtained for Spn-LicC, characterized by a narrow distribution for the cognate ligand CDP-Cho and a broad bimodal distribution of dihedral angles for the non-cognate CMP-AEP. Insofar as dihedral angle distributions represent movement of the ligand, the narrow distribution of cognate ligand conformations may reflect a more tightly bound ternary complex. Although not observed in the crystal structure, it is possible that a proportion of the LicC:ligand complex in solution would carry an additional Mg^2+^ ion, similar to the observed Tde-PntC:CMP-AEP complex. We therefore also performed simulations with a second Mg^2+^ for both Spn-LicC product complexes, revealing a bimodal distribution of the dihedral for CDP-Cho with a second peak around –150° (Supplementary Fig. 16) and a narrower distribution for CMP-AEP. This may explain why Spn-LicC can accept the non-cognate substrate (e.g. with two Mg^2+^ bound), albeit with increased *K*_M_ (Table 1) due to the limited interactions of the primary amine in the LicC choline-binding pocket. Overall, the molecular dynamics simulations provided further support for the influence of steric effects on enzyme selectivity.

### Mechanistic insight from structural data

**Fig. 6:**
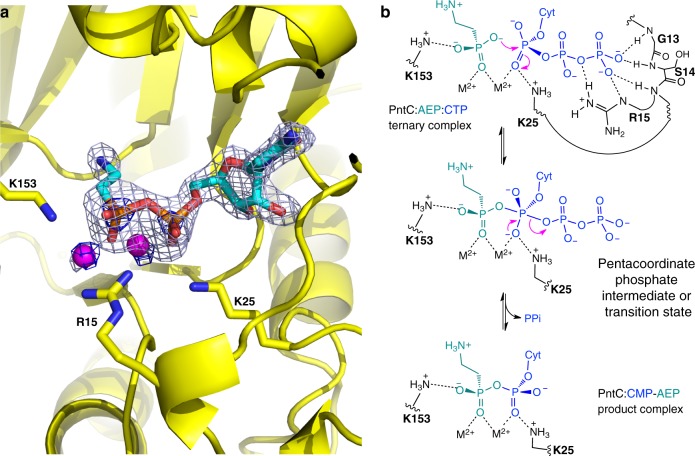
Proposed mechanism of PntC. **a** CMP-AEP electron density (gray mesh: Fo-Fc omit map contoured at 3.5 sigma; blue mesh: 2Fo-Fc map contoured at 5.0 sigma highlighting the heavier Mg and P atoms) for the Tde-PntC:CMP-AEP complex. **b** Mechanistic interpretation of the crystallographic data. The proposed ternary complex structure (top) with extensive interactions stabilizing the PPi leaving group of CTP (blue). Nucleophilic attack of AEP (teal) generates a pentacoordinate phosphate intermediate or transition state possessing additional negative charge stabilized by Lys25 (middle). In the absence of obvious candidate general base residues, we propose that active site p*K*_a_ perturbations may result in the deprotonated AEP substrate shown. Loss of PPi generates the CMP-AEP product bound in the active site (bottom) as seen in the crystal structure in **a**

The crystal structures determined in apo form (PDB 6PD1) and in the presence of CTP and AEP (PDB 6PD2) provide additional mechanistic insight. Electron density outlines the CMP-AEP product (Fig. 6a) with partial density at the expected location of the pyrophosphate (PP_i_) leaving group. This PP_i_ pocket was also detected by the Roll algorithm in both Spn-LicC and Tde-PntC^46^ (Fig. 5a, b). The PP_i_ site is defined by the first five residues of the GXG(T/S)RX_4–8_PK nucleotidyltransferase consensus sequence (boxed region, Supplementary Fig. 15)^32^, which provides stabilizing positive charge via backbone amide protons and the conserved residue Arg15. The terminal Lys25 of the consensus sequence is positioned to interact most strongly with the magnesium-coordinated α-phosphate of CTP, and alanine substitution of this highly conserved residue abolished cytidylyltransferase activity in the related enzymes FrbH (K38A) and YgbP (K27A)^48,49^. Similar to the Spn-LicC structure, Lys25 appears positioned to accept additional negative charge arising during a pentacoordinate transition state, which would result from nucleophilic attack of AEP in an associative mechanism (Fig. 6b). The partially conserved residue Lys153 may guide attack by interacting with phosphonate oxygen. This mechanistic trajectory was also proposed based on a similar crystallographic orientation of Lys27 in the MEP cytidylyltransferase YgbP^49^. Indeed, we observed markedly diminished activities (<10% of wildtype) when alanine was substituted at Arg15, Lys25, and Lys153 of Tde-PntC (Supplementary Fig. 17), lending support to the mechanism proposed in Fig. 6b. Although detailed kinetic analysis was beyond the scope of this study, we expect that PntC enzymes will employ ordered sequential binding. Previous substrate and product inhibition analyses of Spn-LicC favored CTP as the leading substrate and CDP-Cho as the final product off the enzyme^29^, and initial CTP binding was concluded from pulse chase analysis of YgbP^49^.

## Discussion

We have identified nucleotidyltransferase Pfam families in about two-thirds of phosphonate biosynthetic gene clusters. Surprisingly, about 60% of all Ppm enzymes are fused to one or both of two Pfams, with MobA-like NTP transferase (PF12804) as the most commonly fused domain. Only one member of this family was previously known to act on a phosphonate substrate; FrbH catalyzes cytidylyltransfer to L-2-amino-4-phosphonobutyrate during FR-900098 biosynthesis^48^. The other commonly fused domain is cytidylyltransferase-like (PF01467), which includes the PhpF phosphonoformate cytidylyltransferase functioning in phosphinothricin biosynthesis^50^ and the Fom1 HEP cytidylyltransferase from the fosfomycin biosynthetic pathway^28^. In demonstrating AEP-specific cytidylyltransferase activity for two PF12804 enzymes, we have expanded the catalytic repertoire of this family to include phosphonyl tailoring cytidylyltransferases (PntCs) involved in putative cell surface tailoring pathways.

Cytidylyl activation has been well documented in the biosynthesis of cell surface glycans and phospholipids, but is rare in natural product biosynthesis. Indeed, we are not aware of examples beyond small molecule phosphonates and the MEP pathway (e.g. YgbP of the IspD family) for terpenoid biosynthesis. In contrast, CMP-activated sialic, legionaminic, pseudaminic acids, as well as CMP-Kdo, exemplify common intermediates in cell surface glycan biosynthesis^38,51^. Similarly, CMP-conjugates facilitate phospholipid biosynthesis, including CDP-diacylglycerol and the aforementioned CDP-choline^52,53^. The significance of cytidylyltransferase-enrichment in phosphonate biosynthesis is unclear, but may simply reflect that most phosphonates occur on cell surface glycans and lipids.

We expect that PntC enzymes will activate a variety of phosphonates to furnish a mosaic of cell surface phosphonate chemistry. Identifying CMP-conjugates will enable discovery of new phosphonyl modifications and elucidation of their biological roles. Further examples of related chemical logic in biosynthetic pathways, such as AEP and the virulence factor P-Cho, will present opportunities to understand and manipulate enzyme selectivity for developing biocatalysts and selective antivirulence agents.

## Methods

### General

CTP was purchased from Thermo Fisher Scientific (Mississauga, ON, Canada). Chelex 100 sodium form (50–100 mesh), AEP, P-Cho, and CDP-Cho were purchased from Sigma-Aldrich (Oakville, ON, Canada). P-Cho was treated with Chelex 100 resin as previously described prior to use in order to remove calcium, which inhibits the enzyme^32^. All other chemicals were used without further purification. DNA sequencing was performed at The Centre for Applied Genomics at the Hospital for Sick Children (Toronto, ON, Canada). HR–ESI–MS was performed at the Alberta Glycomics Centre (Edmonton, AB, Canada). HPLC analysis was performed using a Prominence LC-20AT system equipped with a diode array detector (Shimadzu, Kyoto, Japan). FPLC purifications were performed using an NGC™ Chromatography System (Bio-Rad, Mississauga, ON). NMR spectra were recorded on an Agilent DD2 operating at 400 MHz for ^1^H and 100 MHz for ^13^C. A Varian Unity Inova operating at 121 MHz was used to record ^31^P NMR spectra.

### Establishing hidden Markov models

Ppm protein sequences were retrieved from NCBI with the following accession numbers: *Tetrahymena pyriformis* (P33182); FrbD from *Streptomyces rubellomurinus* (ABB90393); *Bacillus subtilis* subsp. spizizenii str. W23 (WP_003223692); *Streptomyces viridochromogenes* (AAU00071); *Streptomyces luridus* (ACZ13456); *S. wedmorensis* (BAA32495); *Streptomyces fradiae* (ACG70831); *Pseudomonas syringae* (AFM38986). Cytidylyltransferase (PntC) protein sequences were retrieved from NCBI with the following accession numbers: Tde-PntC from *T. denticola* ATCC 35405 (NP_992021); Oul-PntC from *O. uli* DSM 7084 (ADK67708); Ari-PntC from *A. rimae* ATCC 49626 (ZP_03568201). The retrieved sequences were used to collect representative HMMs for Ppm and putative PntC enzymes from the Pfam database using the HMMER software suite^54,55^. The GA (gathering) score thresholds were applied for determining matches, which is considered a reliable curated threshold for defining family membership^55^.

### Retrieving Ppm and PntC homologs from HMMs

HMMs matching the retrieved Ppm and PntC mentioned above (for Ppm: PF13714; for PntC: PF12804, PF00483, PF01129) were used to scan collectively annotated protein sequences from the NCBI Refseq genome databases^56^ of complete genomes (*n* = 8582), genome scaffolds (*n* = 41,568), and contigs (*n* = 60,795), using HMMscan. After potential homologs were thus identified, a Python script was written and used to narrow the results into homologs sharing alignment to at least one of the HMMs for PntC and the single HMM retrieved for Ppm. To improve accuracy of the search, results were filtered to ensure that only sequences with ≥60% alignment to the HMMs were included. Ppm homologs were further screened to ensure the presence of the conserved catalytic motif EDKXXXXXNS, which distinguishes PEP mutase from other members of the isocitrate lyase family^4^.

### Inventory of Ppm fusion domains

Protein sequences were retrieved for all identified Ppm homologs from NCBI RefSeq genome databases and scanned for HMMs using the HMMER software suite to establish an inventory of fusion domains. The GA (gathering score) threshold was once again applied for determining family membership. A Perl script was applied to account for overlapping domains. In the case of overlapping domains, only the domain with the highest score was included in analysis. Results were also filtered with ≥60% alignment to the HMMs as described above.

### Distribution of distances between ppm and pntC genes

A Python script was written to calculate distances, in number of genes, between *ppm* and *pntC* homologs (after filtering for ≥60% HMM alignment and presence of Ppm motif) in complete, contig, and scaffold NCBI RefSeq genome databases. If multiple *ppm* or *pntC* were present in one genome/contig, only the closest distance was tabulated. To further limit the results to non-redundant genomes (one per species), the genome database was filtered using tri-nucleotide DNA signatures as reported previously^57^. The genomes were filtered at a distance delta = 0.03, which roughly corresponds to species.

### Sequence alignment and homology modeling

The following amino acid sequences were retrieved from NCBI and submitted to the Clustal Omega website for alignment:^58^ Bfr-PntC from *B. fragilis* 638R (CBW22390); Tde-PntC from *T. denticola* ATCC 35405 (NP_992021); Cj1416 from *Campylobacter jejuni* (CAI38904); Oul-PntC from *O. uli* DSM 7084 (ADK67708); Ari-PntC from *A. rimae* ATCC 49626 (ZP_03568201); Spn-LicC from *S. pneumoniae* R36A (AAK94072); Hin-LicC from *Haemophilus influenzae* C486 (AJO89865). Homology models were generated via amino acid sequence submission to the I-TASSER server^59^. Protein pocket sizes were calculated using the Roll algorithm of the web-based POCASA program after deleting the CMP-conjugate ligand but not metal cofactor(s)^46^.

### Molecular dynamics simulations

The crystal structures of 1JYL (chain D) and 6PD2 (focusing on the chain B active site of the BD dimer) were used with experimental coordinates for cognate substrates CDP-Cho and CMP-AEP, respectively. Non-cognate substrates (i.e. 1JYL:CMP-AEP and 6PD2:CDP-Cho) were built in PyMOL^60^. The Enlighten plugin for PyMOL was used to execute protocols using AmberTools14 software for ligand parameterization with GAFF^61^, hydrogen and solvent addition (PREP protocol), structure relaxation and minimization (STRUCT protocol), and molecular dynamics (MD at 300 K; DYNAM protocol)^62^. All simulated active site residues were in their standard protonation states; Tde1415 histidines within the solvated simulation sphere were protonated as follows: HIE57, HID186, HIE187, HIE197, and HIE226. For each of the four complexes, 10 independent 150 ps MD simulations were run using a 20 Å solvent shell surrounding P_A_ atom (equivalent of CTP α-phosphate P) of the substrate. From the 1500 ps of simulation data for each complex, dihedral angles were measured every 1 ps using the cpptraj program included in AmberTools^63^. The simulation data was clustered using cpptraj (hierarchical agglomerative clustering on the RMSD of the substrate, with *ε* = 0.85 for all complexes except for Tde-PntC:P-Cho complex, which used *ε* = 1.1) to identify centroid conformations that were representative of most common conformations observed.

### Bacterial strains and media

*Escherichia coli* BL21(DE3) and *E. coli* DH5α (Supplementary Table 2) from Life Technologies (Carlsbad, CA) were used for protein production and cloning, respectively, and were cultured in standard LB broth. The *spn-licC* gene was purchased from BioBasic Canada (Markham, ON) in the pET57 vector to include the *N*-terminal sequence MGSSH_6_SSGLVPRGSH prior to the *N*-terminal methionine residue and preserve the exact sequence of the previously characterized enzyme^29,33^. The synthetic *spn-licC* gene was directly cloned as an *Nde*I/*Xho*I fragment into pET29 to afford pGH1000. The *ari1348* gene was purchased codon-optimized for *E. coli* from BioBasic in the pET28a vector and subcloned as an *Nde*I/*Not*I fragment into pET29. The resulting pGH2000 expression construct is designed to generate *C*-terminal His_6_-tagged Ari-PntC protein. The *tde1415* gene was purchased from BioBasic in pET28a and subcloned without its stop codon as a *Nde*I/*Not*I fragment into pET21b to afford pGH3000 for producing *C*-terminal histidine-tagged protein. Constructs are summarized in Supplementary Table 3. *A. rimae* (ATCC^®^ 49626^TM^) and *O. uli* ATCC^®^ 49627^TM^ were purchased from Cedarlane Labs (Burlington, ON, Canada) and grown in PYG broth plus 0.1% (v/v) Tween 80 (ATCC^®^ medium 1482) from Anaerobe Systems (Morgan Hill, CA). Anaerobic media for these two organisms was either reduced by incorporating 1 g/L l-cysteine hydrochloride (Fisher Scientific, Hampton, NH) in the media or through incubation (minimum of 24 h) in a Bactron II Anaerobic Chamber (Sheldon Manufacturing Inc., Cornelius, OR) containing an anaerobic gas mixture (consisting of 5% CO_2_, 5% H_2_, and the balance N_2_ supplied by Praxair). *O. uli* and *A. rimae* were grown to stationary phase (~48 h) in the anaerobic chamber at 37 °C in a stationary batch format of 1 L culture volumes.

### RT-PCR analysis

Gene transcription was monitored with primers outlined in Supplementary Table 4. RNA extraction from bacteria was performed using the Ribopure RNA isolation kit (Invitrogen, Carlsbad, CA). Any remaining genomic DNA that may have been carried through the RNA extraction process was removed with a 1 h DNaseI treatment step, which was included in the kit. Following RNA extraction and DNaseI treatment, a DNA-based PCR was always performed to detect undigested DNA. The lack of amplification products confirmed the absence of contaminating genomic DNA. RT-PCR was carried out using the OneStep RT-PCR kit (Qiagen) according to the manufacturer’s recommendations. Briefly, each reaction setup consisted of 2 µl of dNTP mix (400 µM of each dNTP in a reaction), 0.6 µM of each primer, 2 µl of polymerase mix and 2 µl of specific RNA templates. The thermal cycling protocol was performed with the iCycler iQ multicolor real-time detection system (Bio-Rad, Hercules, CA) and contained an initial reverse transcription step of 50 °C for 30 min followed by PCR activation at 95 °C for 15 min. Subsequent steps in the protocol contained 40 cycles of amplification (94 °C for 45 s, 55 °C for 45 s, 72 °C for 1 min) and a concluding step at 72 °C for 10 min. Amplified products were analyzed on 1.8% (w/v) agarose gels stained with 0.5 µg/ml ethidium bromide and imaged with a VersaDocTM 4000 MP (BioRad).

### Site-directed mutagenesis and analysis of mutants

Tde1415 enzyme variants R15A, K25A, and K153 were generated by overlap extension PCR mutagenesis using primers listed in Supplementary Table 4. All mutants were verified by sequencing and proteins expressed in pET21b. Activity of enzyme variants was monitored by ^31^P NMR after 2 h incubation at room temperature in 50 mM Tris–Cl pH 8.0 containing 7.0 mM CTP, 3 mM AEP, 10 µM Tde1415, and 7.0 mM of MgCl_2_. Phosphonoacetic acid at 6.0 mM was added as an internal standard immediately prior to ^31^P NMR spectral acquisition.

### Protein production

Plasmids were transformed into *E. coli* BL21(DE3) and plated on the LB agar plates containing the appropriate antibiotic. Single colonies were picked into ~5 ml and grown overnight at 37 °C in LB supplemented with antibiotic, 1 ml of which was then transferred to 50 ml LB supplemented with antibiotic. After overnight growth at 37 °C, 1 l batches of fresh LB were inoculated with 10 ml of the overnight culture and incubated at 37 °C and 220 rpm. At OD_600_~0.5, cultures were cooled on ice to ~18 °C, supplemented with 0.1 mM IPTG, and then incubated overnight at 18 °C. Cells were harvested by 15 min centrifugation at 4 °C and 4000×*g* and subsequent steps were performed on ice. From 1 L of culture, the resulting pellet was resuspended in cell lysis buffer (50 ml, 0.73 M sucrose, 50 mM Tris–Cl, pH 8.0) and supplemented with 4 mM dithiothreitol (DTT, Amresco, Solon, OH). Egg white lysozyme (1 mg, Amresco) was added and stirred for 10 min, after which deoxycholate solution (50 ml) was added and the solution was stirred until it became viscous (~10 min). The deoxycholate solution contained 24 mM deoxycholate sodium salt (BioBasic, Markham, ON), 0.5% v/v Triton X-100 (BioBasic), 20 mM Tris–Cl, pH 8.0, 0.3 M NaCl. A solution of 1 M MgCl_2_ (500 µl) was added together with deoxyribonuclease I from bovine pancreas (Sigma-Aldrich) and stirred at room temperature until the solution became less viscous. The solution was then centrifuged at high speed (14,000×*g*) for 20 min at 4 °C to afford clarified supernatant raw extract.

### Protein purification

To each 50 ml of raw extract was added 2 ml of Ni-NTA agarose affinity resin (QIAGEN, Hilden, Germany) and gently shaken at 4 °C and 120 rpm for 40 min. The resin was then washed and eluted with 20 and 250 mM imidazole, respectively, in 50 mM Tris–Cl buffer pH 8.0 containing 300 mM NaCl. Eluted protein (10 ml) was dialyzed overnight at 4 °C in 50 mM Tris–Cl, pH 8.0 using MWCO 3500 dialysis tubing, and then run over a HiTrap Q FF anion exchange column (GE Healthcare, Chicago, IL) attached to an NGC FPLC system (Bio-Rad, Hercules, CA). After isocratic flow in 50 mM Tris–Cl (pH 8.0) for 2.5 column volumes, a linear gradient from 0 to 700 mM NaCl was performed over 18 column volumes. Tde1415 eluted at ~120 mM NaCl, and Ari1348 eluted at ~250 mM NaCl, consistent with the respective predicted p*I* values of 5.7 and 5.0 as calculated using the ProtParam tool on the ExPASy web server^64^. Pooled FPLC fractions were concentrated to ~2 ml by ultrafiltration using 10 kDa MWCO centrifugation filters (Pall Corporation, Port Washington, NY), then desalted into Tris–Cl pH 8.0 buffer using PD-10 columns (GE Healthcare). The desalted protein was concentrated to ~200 µl by ultrafiltration, then either used immediately or frozen as beads in liquid nitrogen for storage at −80 °C until further use.

### Protein analysis

Protein concentrations were estimated using the Bradford assay^65^, and purity was assessed using SDS–PAGE. Oligomeric structure was evaluated using size-exclusion chromatography and DLS. The former was performed on a Bio-Rad NGC FPLC system equipped with a Superdex 75 10/300 GL column from GE Healthcare, eluted using an isocratic flow (0.5 ml/min) of 50 mM Tris–Cl and 150 mM MgCl_2_ at pH 8.0. DLS was performed on 20 µl of freshly purified protein (8 mg/ml) at 20 °C using a DynaPro NanoStar (Wyatt Technology, Santa Barbara, CA).

### HPLC enzyme assays

Reactions containing 50 nM enzyme, 1 mM of either AEP or P-Cho, 4 mM CTP, 7 mM MgCl_2_, and 50 mM Tris–Cl pH 7.8 were allowed to proceed for 30 min prior to quenching a 10 µl aliquot with an equal volume of cold methanol (containing 0.1% TFA). The quenched reaction was centrifuged for 5 min prior to injecting 1 µl on an HPLC equipped with a Luna NH2 column (3 µm, 100 Å, 100 × 4.6 mm, Phenomenex, Torrance CA, USA) using an isocratic elution at 1 ml/min in a mobile phase of 20 mM ammonium acetate, pH 10.

### Aminotransferase activity assays

The AEPT activity was observed by combining 1.5 mM AEP, 6 mM pyruvate, 30 µM pyridoxal-5′-phosphate (PLP) in 50 mM Tris–Cl buffer containing 50 mM MgCl_2_, pH 8.0. The reaction was initiated by adding 1 µM of Tde1415 enzyme, which was missing in the no-enzyme negative control. The reaction was either monitored directly via ^31^P NMR as shown in Supplementary Fig. 11 or allowed to proceed at room temperature for 2 h prior to removal of enzyme by ultrafiltration using a Vivaspin 5000 Da MWCO membrane centrifugation device (Sartorius, Goettingen, Germany). Filtrate was stored frozen at −20 °C prior to mass spectrometric analysis at the Mass Spectrometry Facility at the University of Guelph (Supplementary Fig. 12).

### Metal use assays

140 µl of 400 µM recombinant wildtype Tde1415 was diluted to a volume of 3.0 ml with 50 mM EDTA, 50 mM Tris–HCl, pH 8.0 buffer. The protein solution was applied to an Econo-Pac 10DG desalting column (Bio-Rad) and exchanged into 50 mM EDTA, 50 mM Tris–Cl, pH 8.0 buffer. The eluted protein was concentrated using a 5 kDa MWCO ultracentrifuge device (Sartorius) and then desalted a second time into 50 mM Tris–Cl, pH 8.0. The de-metalated protein was again concentrated to 2.0 ml by ultracentrifugation and stored at 4 ^o^C. PntC activity was monitored by ^31^P NMR after 2 h incubation at room temperature in 50 mM Tris–Cl pH 8.0 containing 7.0 mM CTP, 3 mM AEP, 5.5 µM Tde1415, and 7.0 mM of divalent metal (MgCl_2_, CaCl_2_, or ZnCl_2_). Phosphonoacetic acid at 6.0 mM was added as an internal standard immediately prior to ^31^P NMR spectral acquisition.

### Colorimetric coupled enzyme assays

The EnzCheck^®^ Pyrophosphate Assay Kit (Molecular Probes, Inc., Eugene, OR, USA) was used to determine the activity of Spn-LicC, Ari-PntC, and Tde1415. Each reaction was kept to a total volume of 100 µl and performed in triplicate. The reaction components were split between two wells, one well contained the substrate and enzyme, while the other well contained CTP. Well 1 contained 0.5 µl (1U) PNP, 0.5 µl (0.03U) IPP, 10 µl (0.2 mM) MESG, 2.5 µl 20X reaction buffer, 16 µl (4 mM) CTP, and 20.5 µl of water, making a total of 50 µl. Well 2 contained 0.5 µl (1U) PNP, 0.5 µl (0.03U) IPP, 10 µl (0.2 mM) MESG, 2.5 µl 20X reaction buffer, 12 µl (7 mM) MgCl_2_, enzyme (variable), substrate (variable), and H_2_O to fill to a final volume of 50 µl. A control with either no enzyme or substrate was made for each trial. The separate wells were incubated for 30 min at room temperature. After 30 min, well 2 was combined to well 1, and absorbance was recorded every 30 s at 360 nm for 30 min. Initial velocities (<10% substrate turnover) for each substrate concentration were exported to Excel and Michaelis–Menten and substrate inhibition equations were fit by non-linear regression using the R software^66^.

### Purification of CMP-AEP

5 ml reactions containing 1 µM Ari-PntC, 1 mM of AEP, 2 mM CTP, 7 mM MgCl_2_, and 50 mM Tris–Cl pH 8 were allowed to proceed for 1 h at room temperature prior to removing the enzyme through centrifugation in a 5 kDa ultracentrifugation tube. The flow-through was injected on the FPLC over a 5 ml GE Hi-Trap™ Q FF anion exchange column into the mobile phase A (25 mM ammonium bicarbonate pH 9) and eluted using a linear gradient of 0–70% mobile phase B (25 mM ammonium bicarbonate + 1 M NaCl pH 9). Peak fractions pertaining to the CMP-AEP product were collected and lyophilized to a dry solid. 20–25 mg of the lyophilized product was dissolved into 600 µl of D_2_O before subsequent ^1^H, ^13^C, and ^31^P experiments were performed. Phosphonoacetic acid was added as an internal standard for the ^31^P experiments.

### Protein crystallization

Purified Tde1415 in 50 mM Tris–Cl, pH 8.0 and 240 mM NaCl, were concentrated to 20 mg/ml with and without presence of 5 mM CTP/5 mM AEP. Tde1415 was initially screened against commercially available sparce-matrix screens in a 1:1 ratio, 1 μL final volume using an ARI Crystal Gryphon (Art Robins Instruments). The microplates were sealed, incubated at 18 ^o^C, and conditions in which crystal growth was observed were used to design hanging-drop vapor diffusion expansion crystal plates. The ligand-free structure was determined by crystals grown against a crystallization solution composed of 0.01 M nickel II chloride hexahydrate, 0.1 M Tris–Cl, pH 8.5, 1.0 M lithium sulfate monohydrate and cryoprotected with an equivalent solution supplemented with 30% (v/v) glycerol. The product complex was determined from a crystal grown against a crystallization solution composed of 0.2 M magnesium acetate tetrahydrate, 0.1 M sodium cacodylate trihydrate pH 6.5, 20% (w/v) polyethylene glycol 8000 cryoprotected with an equivalent solution supplemented with 30% (v/v) glycerol.

### X-ray data collection and processing

Protein crystals were retrieved, briefly introduced in cryoprotectant, and snap-frozen in liquid nitrogen. Data analysis was performed at the Advanced Photon Source (APS) at Argonne National Laboratory (Argonne, Illinois, Illinois & Michigan Canal State Trail, 9700 Cass Ave, Lemont, IL 60439, USA) and the Canadian Light Source (CLS) at the University of Saskatchewan (Saskatoon, Saskatchewan, Canadian Light Source Inc., 44 Innovation Boulevard, SK S7N 2V3, Canada). At the APS, X-ray diffraction data was collected at beamline 17-ID of the Argonne National Laboratory Synchrotron Facility. At the CLS, X-ray diffraction data was collected at beamline CLS-08-ID. In both cases images were collected using 0.25 oscillations at a temperature of 100 K and wavelength of 0.9795 nm. Data were indexed and scaled using AutoProcess in the space group P2_1_ and data processed to 2.72 Å resolution. Note that while both datasets belong to the P2_1_ space group, the unit cell dimensions were different. The “apo” structure of Tde1415 was solved via molecular replacement using the program PHASER^67^ searching for four copies of PDB coordinates 1JYK^29^ and 1VJO^37^ which are the cytidyltransferase LicC from *S. pneumoniae* and an alanine-glyoxylate aminotransferase from *Anabaena* [*Nostoc*.] sp. PCC 7120, respectively. Cycles of iterative model building, structure refinement, and density modification were performed with PHENIX^68^ and were interspersed with inspection and manual building with COOT^69^. Restraints for CDP-Cho were generated via the Grade Web Server (http://grade.globalphasing.org). Ramachandran analysis of the apo structure revealed: 2288 (94.1%) torsion angles in the preferred region, 119 (4.9%) allowed, and 25 (1.0%) disallowed; for the Tde1415:CMP-AEP complex: 2325 (95.1%) preferred, 98 (4.0%) allowed, 21 (0.9%) disallowed.

### Reporting summary

Further information on research design is available in the Nature Research Reporting Summary linked to this article.

## Supplementary information

Supplementary Information

Reporting Summary

Source Data

## Data Availability

The authors declare that the data supporting the findings of this study are available within the paper and its supplementary information files of in a public repository. Specifically, associated raw data has been provided as a source data file for Supplementary Figs. 1, 2, 9, 14, and 17. The coordinates and structure factors of Tde1415 and Tde1415:CMP-AEP have been deposited in the Protein Data Bank with respective PDB accession codes 6PD1 and 6PD2. Other data are available from the corresponding author upon reasonable request.

## Code availability

Bioinformatic scripts are available at GitHub: http://github.com/papi0310/ppm-pntC-clustering
