# Supplementary material for: The predominance of nucleotidyl activation in bacterial phosphonate biosynthesis
Source: Nat Commun. 2019 Aug 16;10:3698. doi: 10.1038/s41467-019-11627-6 (PMC6697681; doi:10.1038/s41467-019-11627-6)
Supplement: Supplementary file 1 — Supplementary Information [file 41467_2019_11627_MOESM1_ESM.pdf]

Supplementary Information

The predominance of nucleotidyl activation in phosphonate biosynthesis

K. Rice *et al.*

Supporting Information

**Supplementary Figure 1.**	RT-PCR analysis to detect gene expression	S3
**Supplementary Figure 2.**	SDS-PAGE analysis of recombinant proteins	S4
**Supplementary Figure 3.**	Dynamic light scattering analysis	S5
**Supplementary Figure 4.**	HR-ESI-MS	S6
**Supplementary Figure 5.**	^{1}H NMR spectrum of CMP-AEP	S7
**Supplementary Figure 6.**	^{13}C NMR spectrum of CMP-AEP	S8
**Supplementary Figure 7.**	^{31}P NMR spectrum of CMP-AEP	S9
**Supplementary Figure 8.**	Enzyme activity towards CTP, ATP, and GTP	S10
**Supplementary Figure 9.**	Steady-state kinetic data	S11
**Supplementary Figure 10.**	Overall structure of apo- and holo-Tde1415	S12
**Supplementary Figure 11.**	Transaminase activity of Tde1415 by ^{31}P NMR	S13
**Supplementary Figure 12.**	Transaminase activity of Tde1415 by mass spectrometry	S14
**Supplementary Figure 13.**	Proposed mechanism of AEPT domain of Tde1415	S15
**Supplementary Figure 14.**	Comparison of PntC activity with different metals	S16
**Supplementary Figure 15.**	Multiple sequence alignment of cytidylyltransferases	S17
**Supplementary Figure 16.**	Spn-LicC MD simulations with a second Mg^{2+} ion	S19
**Supplementary Figure 17.**	Analysis of Tde1415 mutants R15A, K25A, and K153A	S20
**Supplementary Table 1.**	X-ray data collection and refinement statistics	S21
**Supplementary Table 2.**	Strains	S22
**Supplementary Table 3.**	Plasmids	S22
**Supplementary Table 4.**	Primers	S23
**Supplementary References**		S24

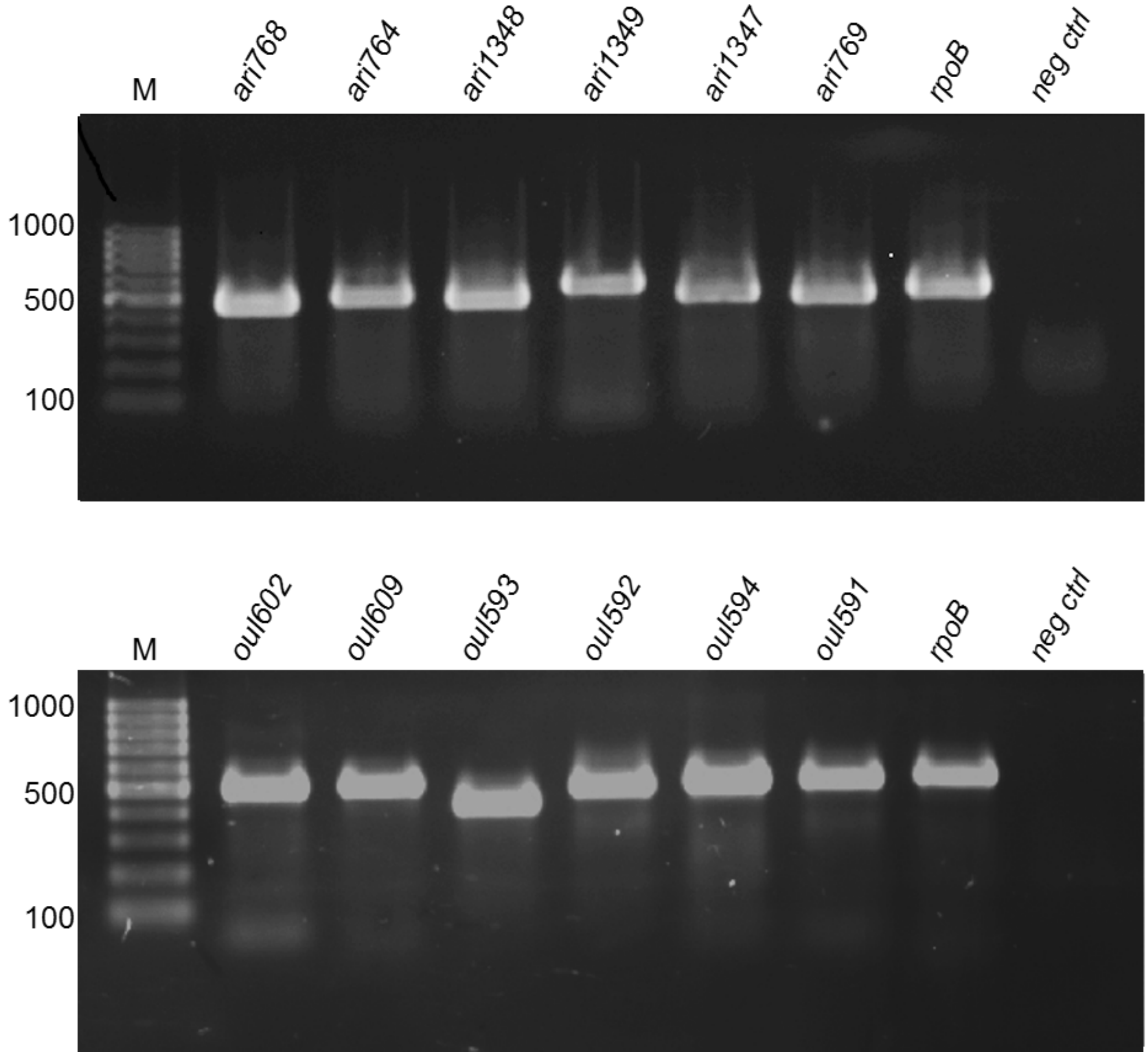

Supplementary Figure 1. RT-PCR analysis demonstrates expression of genes from clusters shown in Figure 3 for *A. rimae* (top) and *O. uli* (bottom). The *rpoB* gene was used as a positive control.[1] Primers used are listed in Supplementary Table 4.

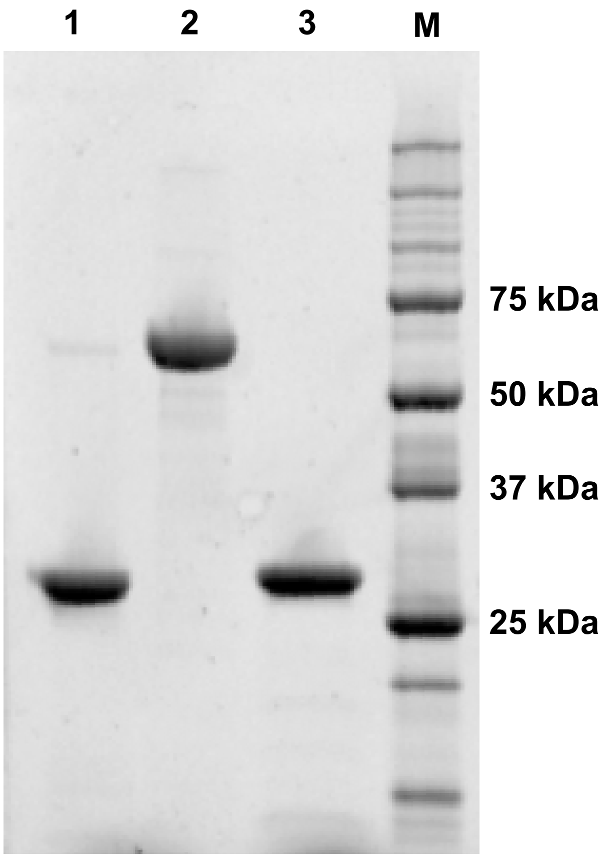

Supplementary Figure 2. SDS-PAGE of Spn-LicC (lane 1), Tde1415 (lane 2), Ari1348 (lane 3), and Bio-Rad Precision Plus Protein Standard (lane M).

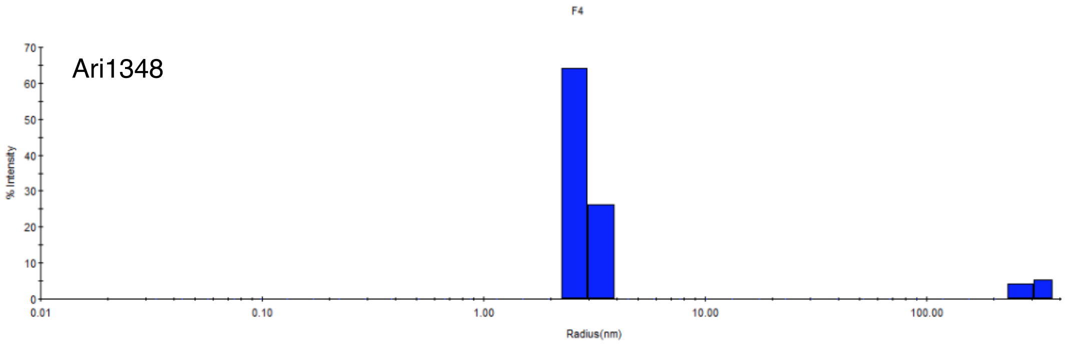

Intensity Distribution	Radius (nm)	%Pd	Mw-R (kDa)	%Intensity	%Mass
Peak 1	2.830	13.0	38	90.4	100.0
Peak 2	313.323	13.2	2329710	9.6	0.0

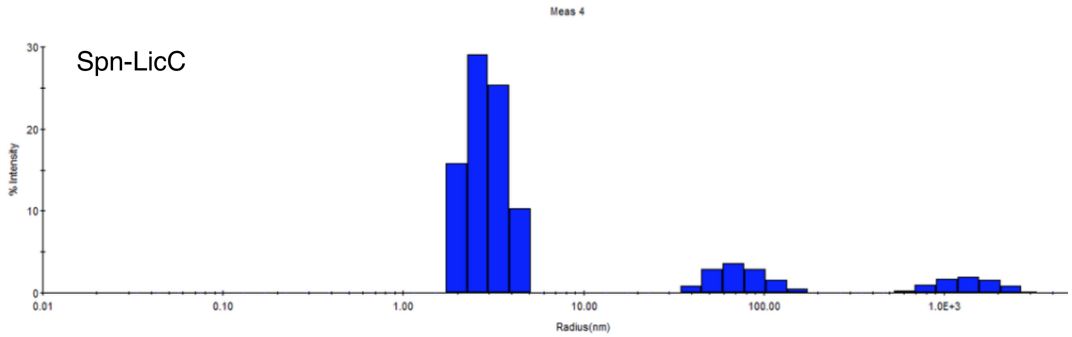

Intensity Distribution	Radius (nm)	%Pd	Mw-R (kDa)	%Intensity	%Mass
Peak 1	2.967	26.0	43	80.4	99.9
Peak 2	77.675	36.5	89138	12.3	0.0
Peak 3	1425.910	38.3	80746700	7.3	0.1

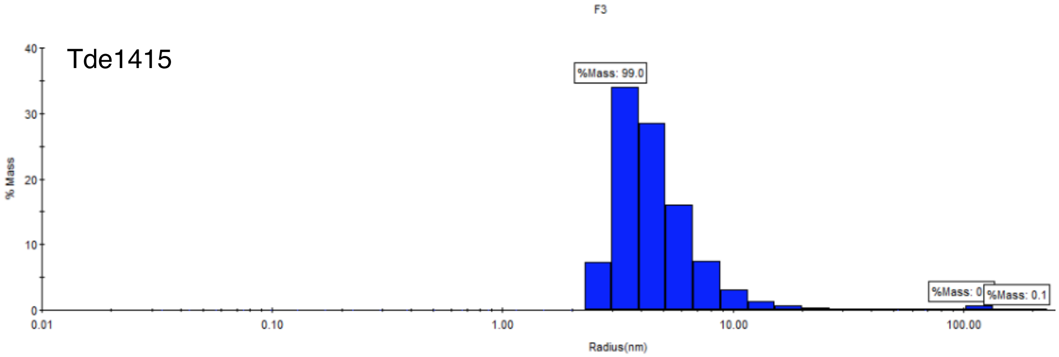

Mass Distribution	Radius (nm)	%Pd	Mw-R (kDa)	%Intensity	%Mass
Peak 1	5.011	68.5	146	80.2	99.0
Peak 2	118.041	13.5	237311	17.9	0.9
Peak 3	205.078	5.4	864189	1.8	0.1

Supplementary Figure 3. Dynamic light scattering data showing molecular weights of 38 kDa for Ari1348, 43 kDa for Spn-LicC, and 146 kDa for Tde1415.

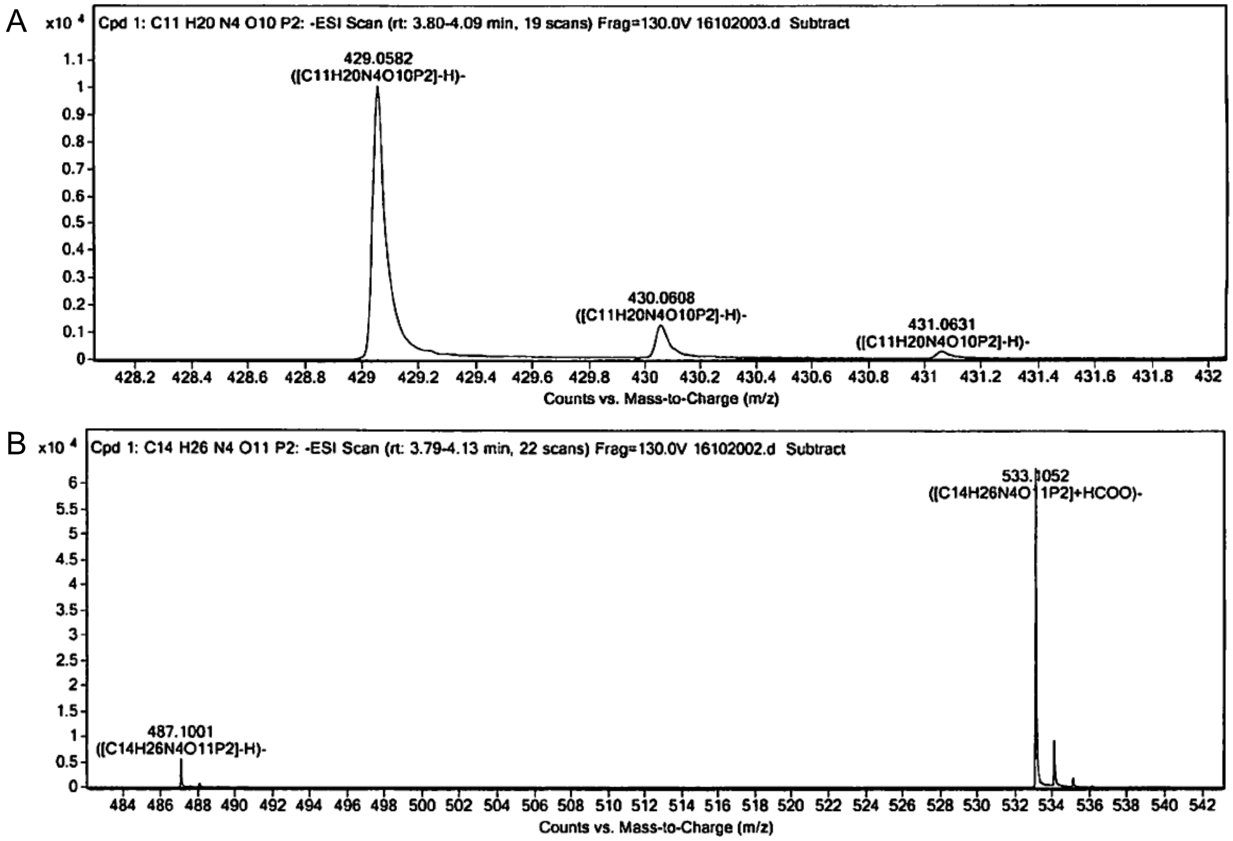

Supplementary Figure 4. HR-ESI-MS of (A) CMP-AEP revealing the $[M-H]^-$ ion at *m/z* 429.0582 consistent with a molecular mass of 430.0655 for molecular formula $C_{11}H_{20}N_4O_{10}P_2$ (calculated 430.0655), and (B) CDP-Cho revealing the $[M-H]^-$ ion at m/z 487.1001 consistent with a molecular mass of 488.1071 (calculated 488.1073).

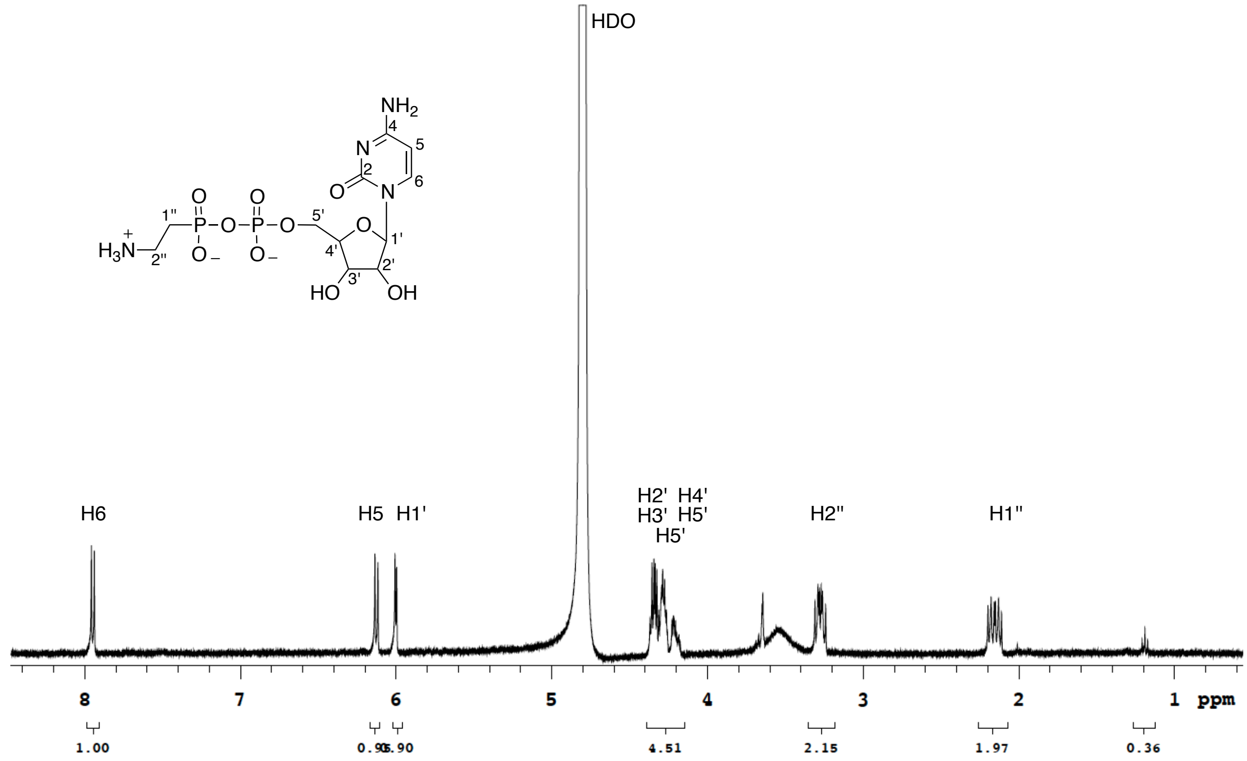

Supplementary Figure 5. ^{1}H NMR (D_2O, 400 MHz) spectra of CMP-AEP: δ 7.95 (d, 1H, H6, J = 7.6 Hz), 6.12 (d, 1H, H5, J = 7.6 Hz), 6.00 (d, 1H, H1', J = 4.0 Hz), 4.82-4.18 (m, 5H, H2', H3', H4', H5'), 3.29 (m, 2H, H2"), 2.15 (m, 2H, H1")

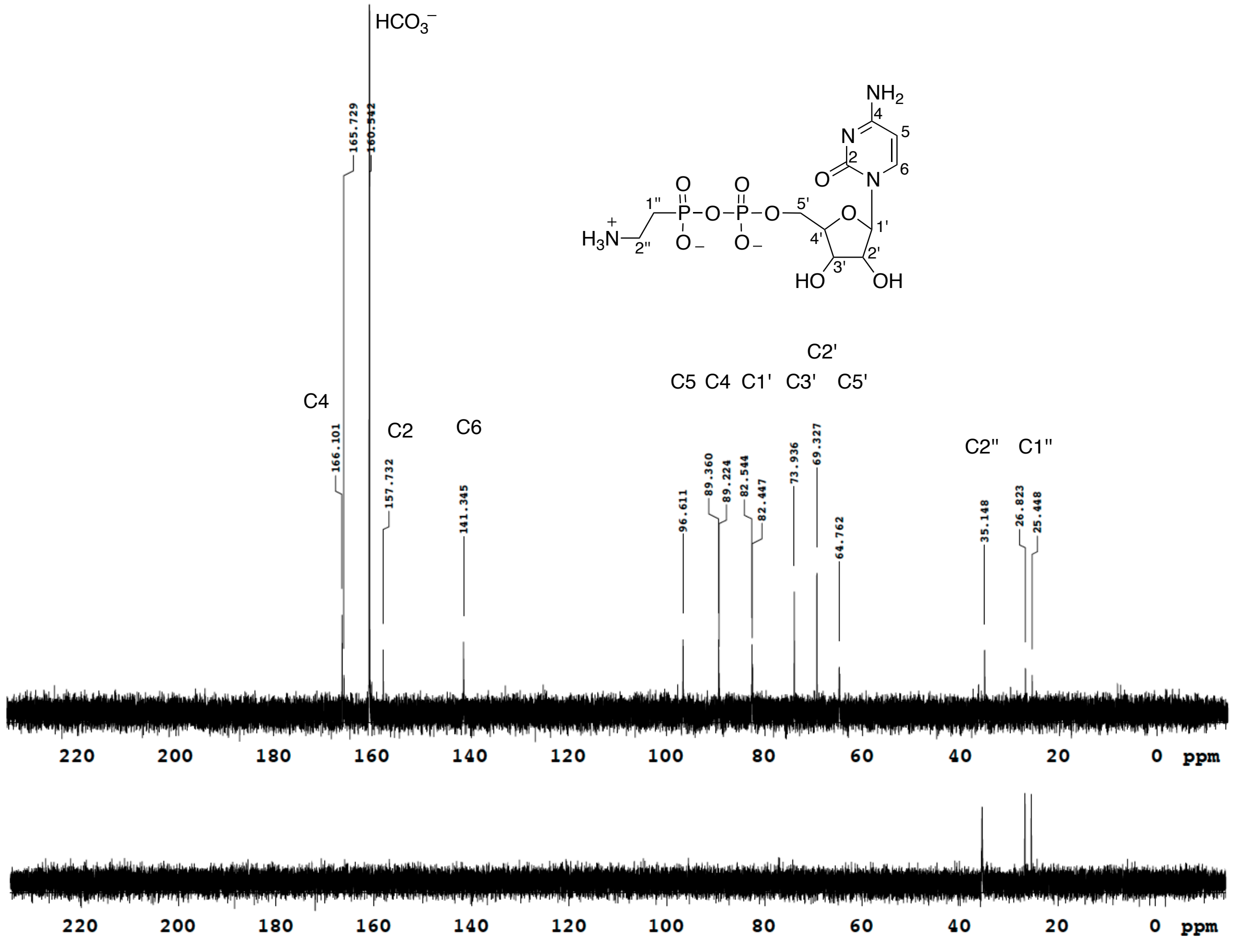

Supplementary Figure 6. ^{13}C NMR (D_2O, 100 MHz) spectra of CMP-AEP: δ 166.1 (C4), 157.7 (C2), 141.3 (C6), 96.6 (C5), 89.3 (C4'), 82.5 (C1'), 73.9 (C3'), 69.3 (C2'), 64.8 (C5'), 35.1 (C2''), 26.1 (C1''; $^{1}J_{CP}$ = 130 Hz). Bottom spectrum represents commercial AEP alone.

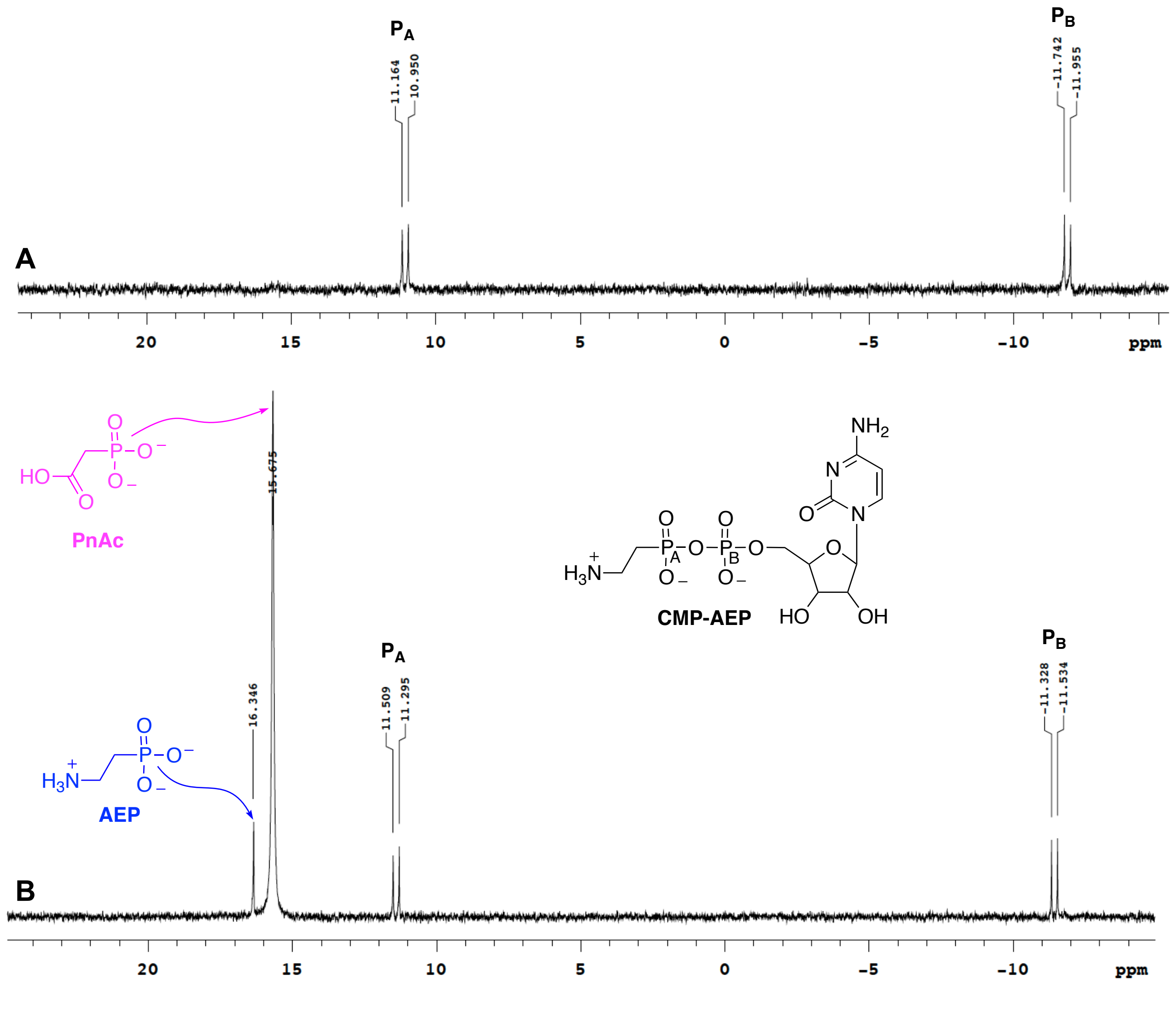

Supplementary Figure 7. ^{31}P NMR (D_2O, 121 MHz) spectra of (A) CMP-AEP alone and (B) with phosphonoacetic acid (PnAc) and 2-ethylaminophosphonate (AEP). Using PnAc as the internal standard with a reported chemical shift of 15.7,[2] our measured chemical shifts of CMP-AEP: δ 11.4 (P_A), -11.4 (P_B), ^3J = 25.6 Hz.

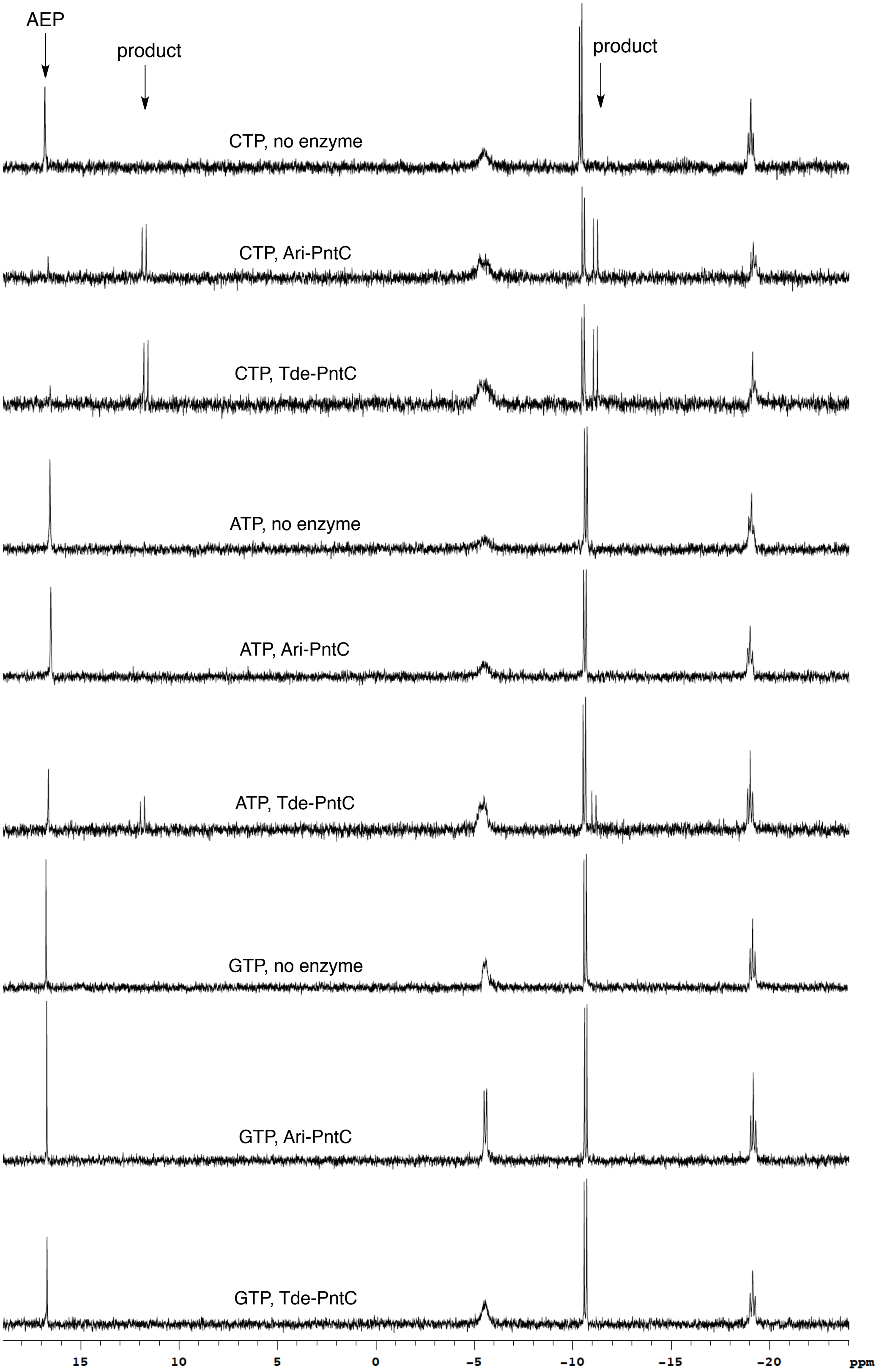

Supplementary Figure 8. ^{31}P NMR analysis of 1-hour reactions of 10 μM PntC enzyme with 1 mM AEP and 2 mM of CTP, ATP, or GTP in 50 mM Tris-Cl and 7 mM $MgCl_2$, pH 8.0.

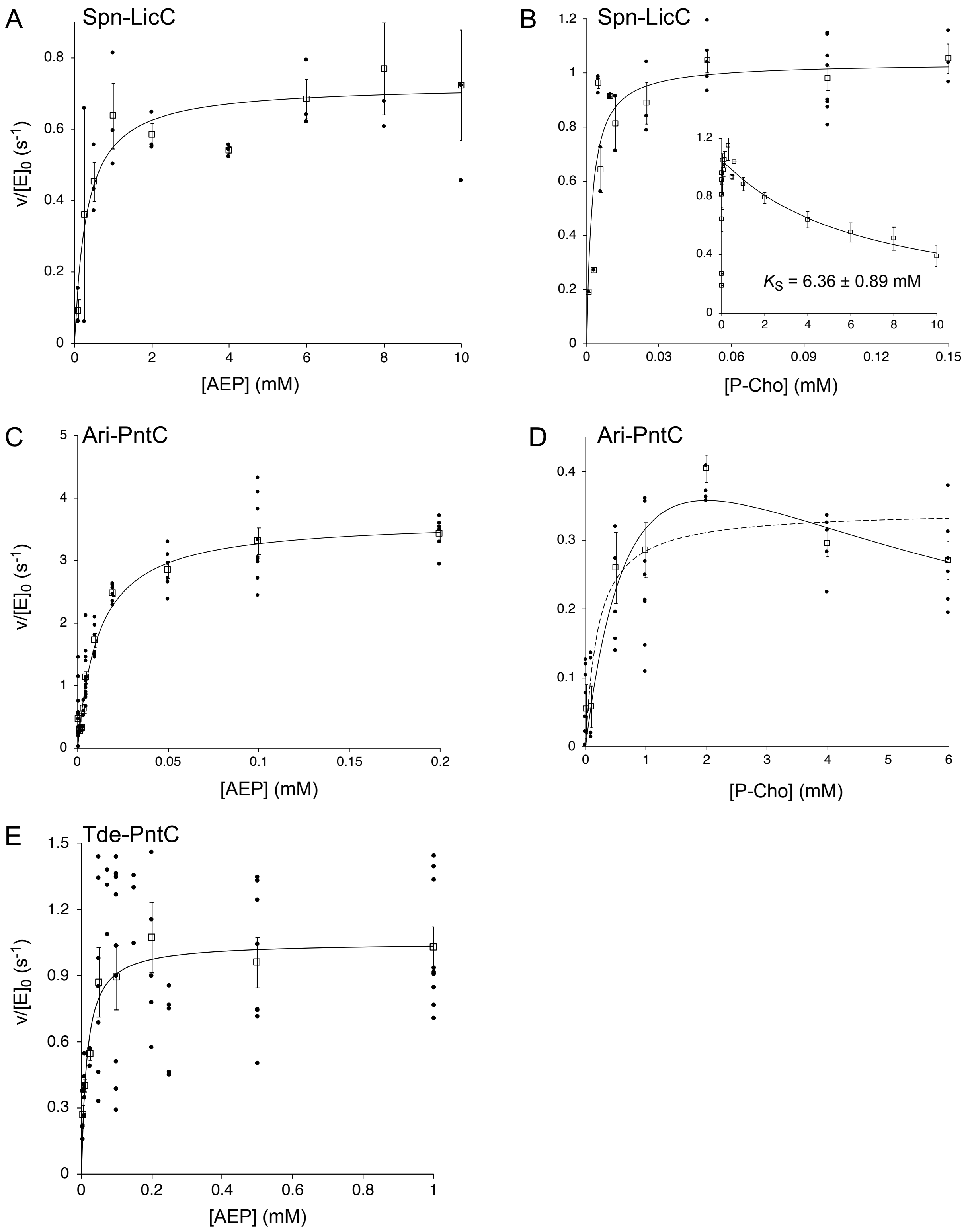

Supplementary Figure 9. Steady-state kinetic analysis of cytidylyltransferase activities of (A) Spn-LicC with AEP, (B) Spn-LicC with P-Cho, (C) Ari-PntC with AEP, (D) Ari-PntC with P-Cho, and (E) Tde-PntC with AEP. Activity not detected for Tde-PntC and P-Cho. Error bars show standard errors of the mean (open square), with all data points shown as filled circles.

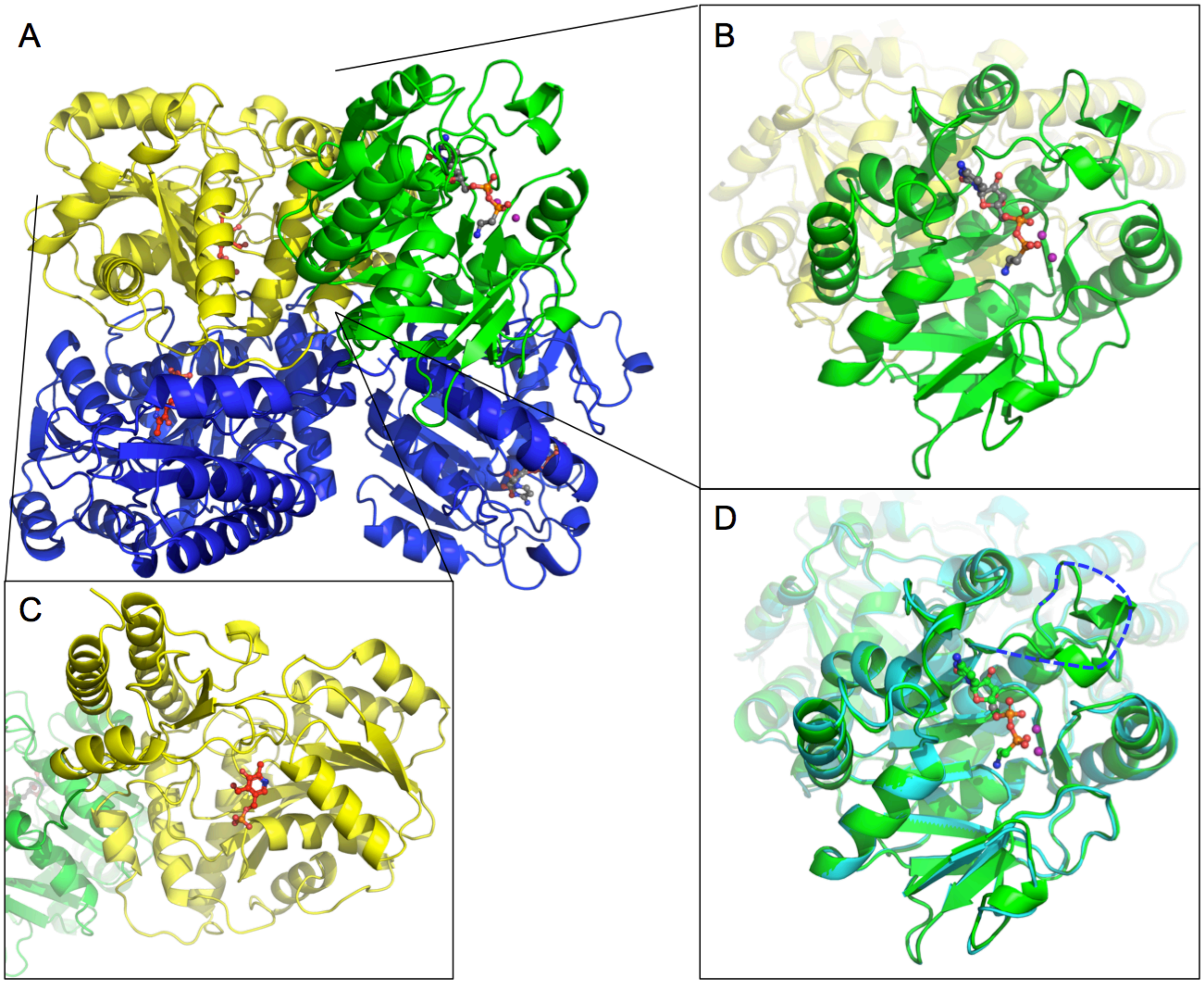

Supplementary Figure 10. Cartoon representations of Tde1415 crystal structures. (A) Overall dimeric stucture showing molecule A in blue and molecule B is bi-colored yellow (residues 1-249) and green (residues 250-615). (B) Tde-PntC cytidylyltransferase domain (green) in complex with CMP-AEP. (C) AEPT domain (yellow) complexed with PLP. (D) Superposition of Tde-PntC-apo (cyan) and Tde-PntC:CMP-AEP (green) reveals low RMSD ranging from 0.53 to 0.86 Å. The exception is the disordered residues 14-20 of Tde-PntC-apo (dotted blue line).

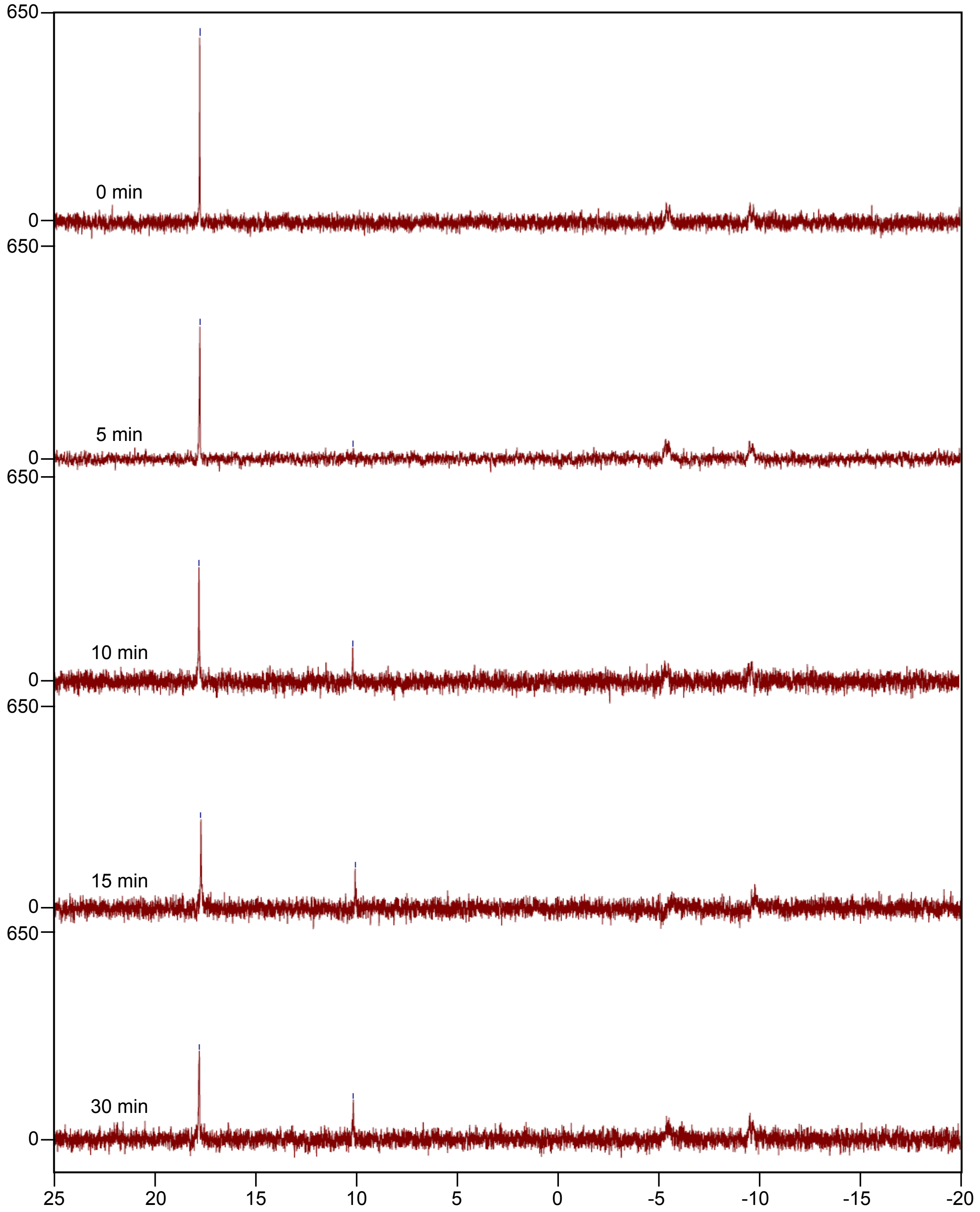

Supplementary Figure 11. Time-dependent conversion of 1.5 mM AEP to PnAA catalyzed by 50 nM Tde1415 in the presence of 6 mM pyruvate, 30 µM PLP, and 300 mM NaCl in Tris-Cl, pH 8. Reactions were run at 20 °C and quenched with an equal volume of methanol at each time point.

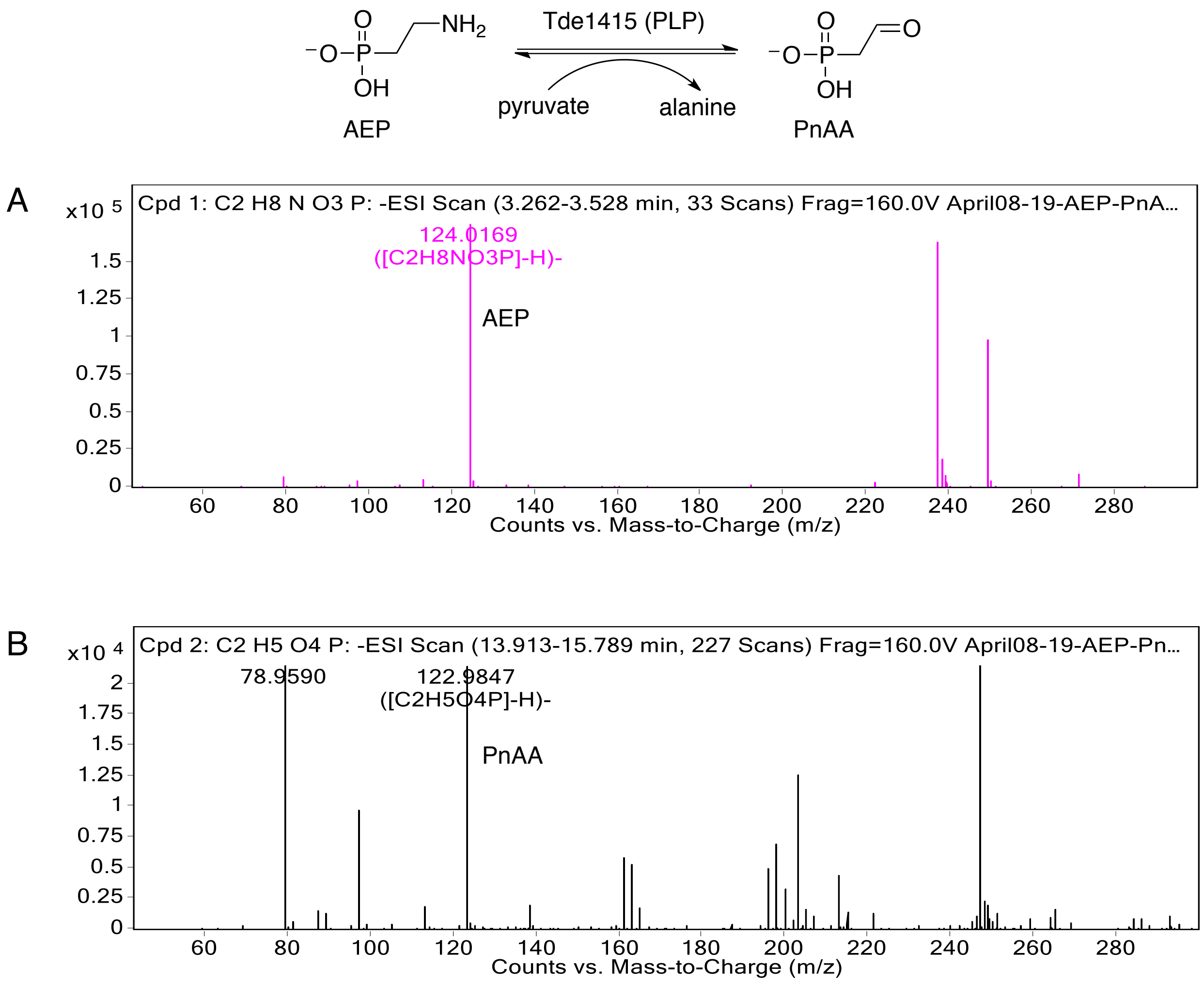

Supplementary Figure 12. Mass spectrometry revealing the aminotransferase activity of Tde1415 as shown. A) Detection of AEP (calculated mass = 124.0169) in the presence of Tde1415 (also observed in no enzyme control) and B) detection of PnAA (calculated mass = 122.9853).

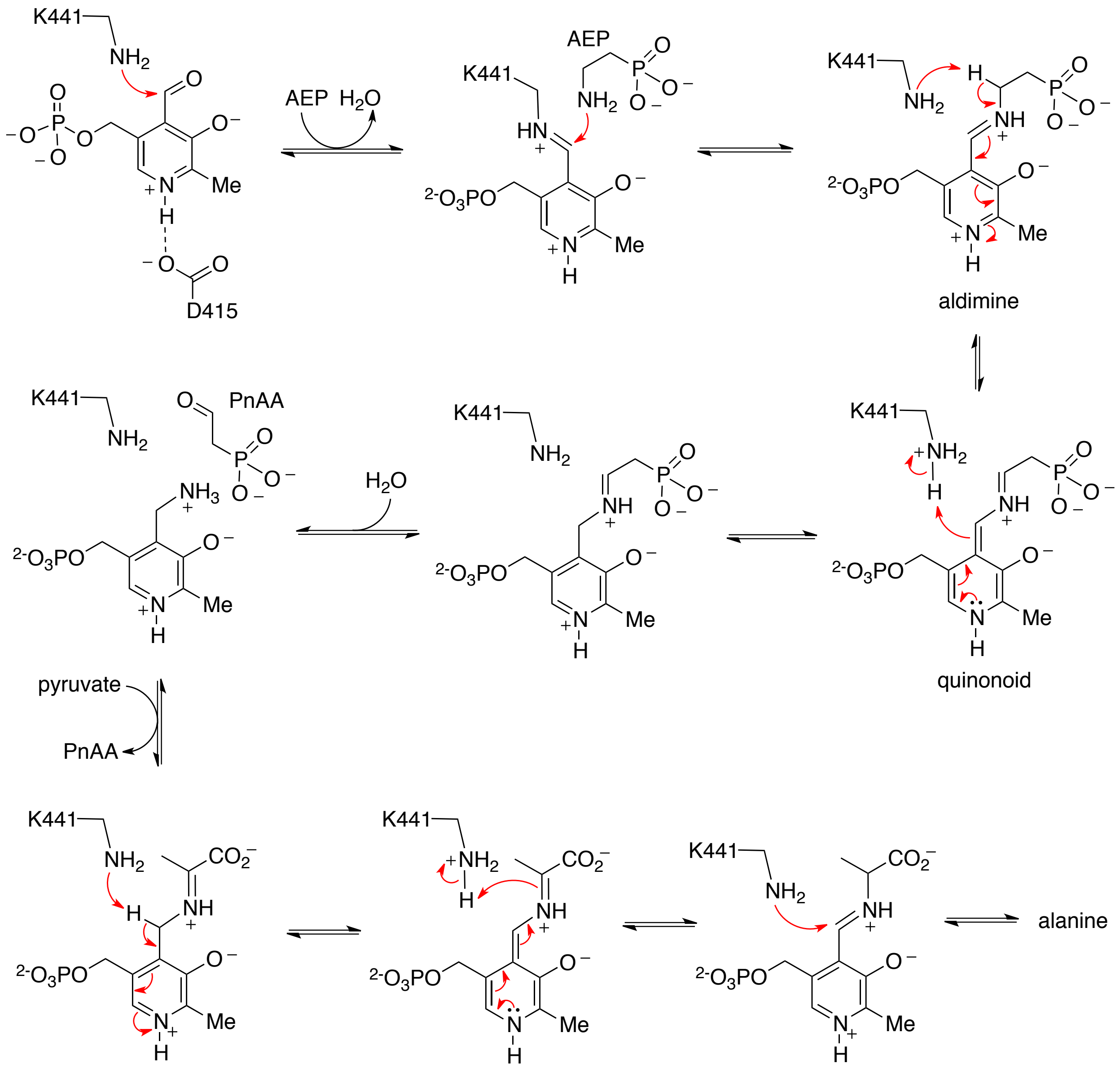

Supplementary Figure 13. Proposed mechanism for aminotransferase activity of Tde1415 catalyzing the interconversion of AEP and PnAA. The first structure represents the crystallographically observed state, which unusually does not have an imine linkage between K441 and PLP.

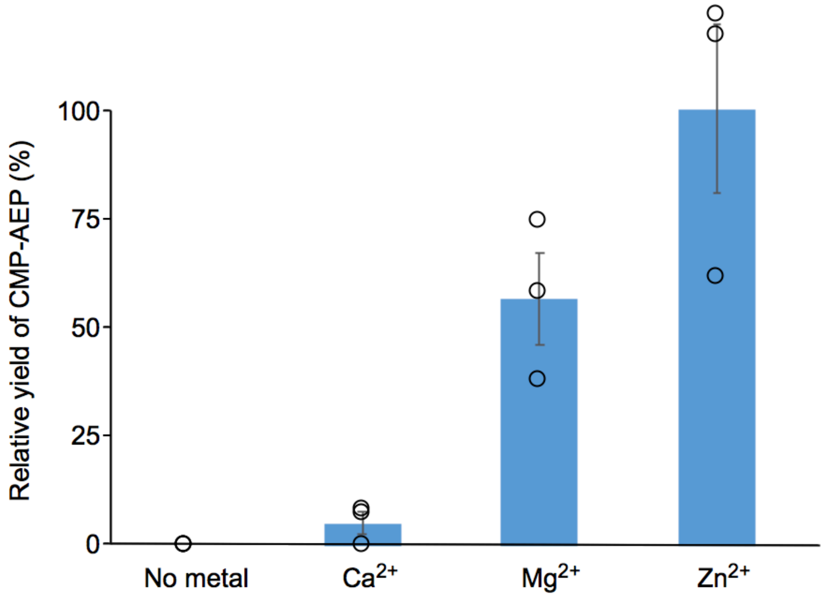

Supplementary Figure 14. Comparison of PntC activity in the presence of different metals. The activity of EDTA-treated enzyme ("No metal") was compared against the same enzyme supplemented with Ca^{2+}, Mg^{2+}, or Zn^{2+}. All four treatments were repeated in triplicate with and are reported with standard errors. Assays were carried out in 50 mM Tris-Cl pH 8.0 with 7.0 mM metal, 7.0 mM CTP, 3.0 mM AEP, and 5.5 µM enzyme. Reactions were allowed to proceed at 20 °C for 2 h, then an internal standard (6.0 mM phosphonoacetic acid) was added immediately prior to acquiring ^{31}P NMR spectra. Peak integrations for CMP-AEP were calculated relative to the internal standard. Bars represent mean values with standard errors shown. Individual data points (three for each metal) are shown as open circles. The yield of CMP-AEP product is assigned as 100% for the most active condition (Zn^{2+}).

A

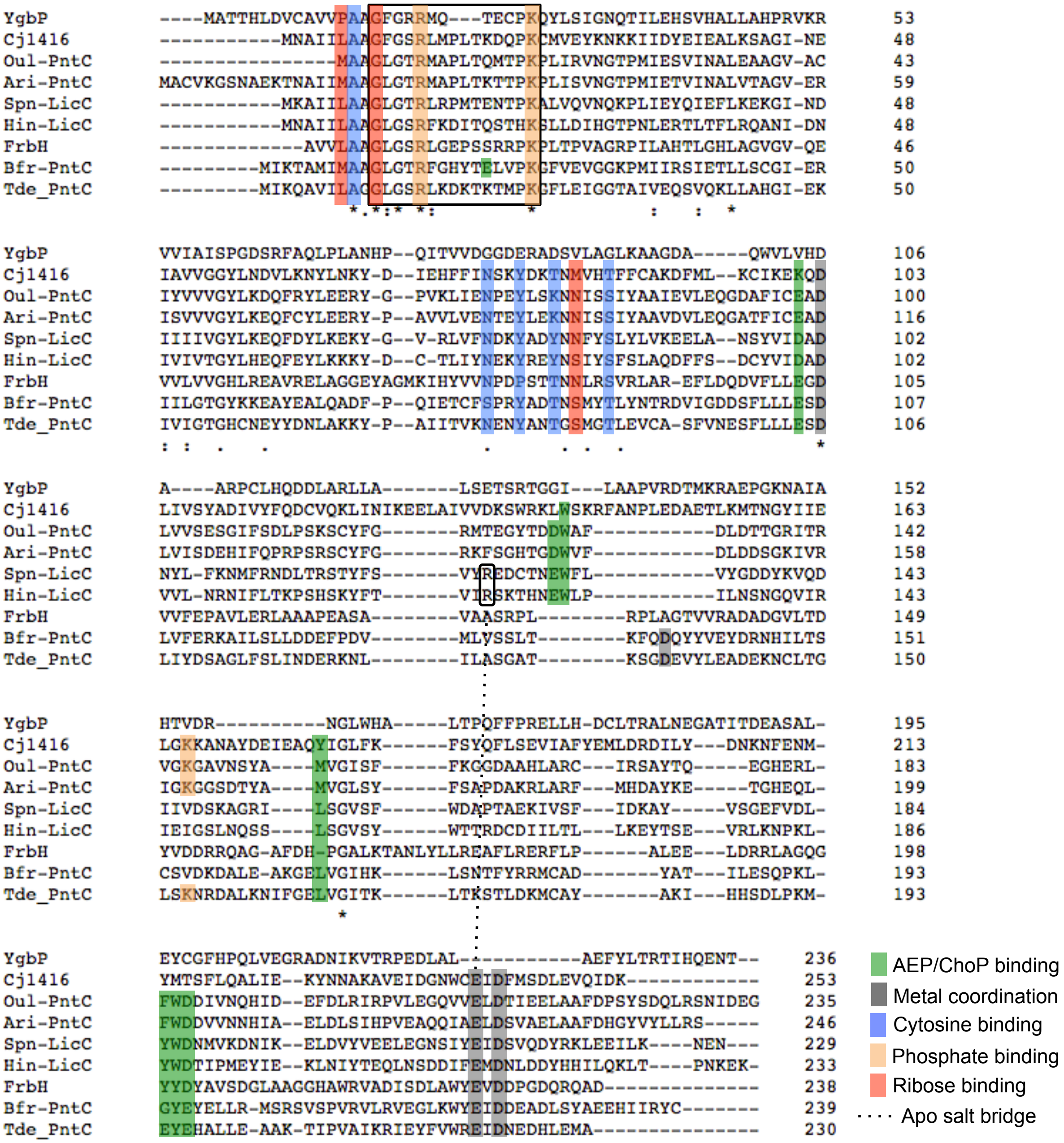

YgbP ----MATTHLDVCAVVPAAGFGRRMQ---TECPKQYLSIGNQTILEHSVHALLAHPRVKR 53
Cj1416 -----------MNAIILAAGFGSRLMPLTKDQPKCMVEYKNKKIIDYEIEALKSAGI-NE 48
Oul-PntC ----------------MAAGLGTRMAPLTQMTPKPLIRVNGTPMIESVINALEAAGV-AC 43
Ari-PntC MACVKGSNAEKTNAIIMAAGLGTRMAPLTKTTPKPLISVNGTPMIETVINALVTAGV-ER 59
Spn-LicC -----------MKAIILAAGLGTRLRPMTENTPKALVQVNQKPLIEYQIEFLKEKGI-ND 48
Hin-LicC -----------MNAIILAAGLGSRFKDITQSTHKSLLDIHGTPNLERTLTFLRQANI-DN 48
FrbH -------------AVVLAAGLGSRLGEPSSRRPKPLTPVAGRPILAHTLGHLAGVGV-QE 46
Bfr-PntC ---------MIKTAMIMAAGLGTRFGHYTELVPKGFVEVGGKPMIIRSIETLLSCGI-ER 50
Tde_PntC ---------MIKQAVILAGGLGSRLKDKTKTMPKGFLEIGGTAIVEQSVQKLLAHGI-EK 50
.:* *: * : : *
YgbP VVIAISPGDSRFAQLPLANHP--QITVVDGGDERADSVLAGLKAAGDA-----QWVLVHD 106
Cj1416 IAVVGGYLNDVLKNYLNKY-D--IEHFFINSKYDKTNMVHTFFCAKDFML--KCIKEKQD 103
Oul-PntC IYVVVGYLKDQFRYLEERY-G--PVKLIENPEYLSKNNISSIYAAIEVLEQGDAFICEAD 100
Ari-PntC ISVVVGYLKEQFCYLEERY-P--AVVLVENTEYLEKNNISSIYAAVDVLEQGATFICEAD 116
Spn-LicC IIIIVGYLKEQFDYLKEKY-G--V-RLVFNDKYADYNNFYSLYLVKEELA--NSYVIDAD 102
Hin-LicC IVIVTGYLHEQFEYLKKKY-D--C-TLIYNEKYREYNSIYSFSLAQDFFS--DCYVIDAD 102
FrbH VVLVVGHLREAVRELAGGEYAGMKIHYVVNPDPSTTNNLRSVRLAR-EFLDQDVFLLEGD 105
Bfr-PntC IILGTGYKKEAYEALQADF-P--QIETCFSPRYADTNSMYTLYNTRDVIGDDSFLLLESD 107
Tde_PntC IVIGTGHCNEYYDNLAKKY-P--AIITVKNENYANTGSMGTLEVCA-SFVNESFLLLESD 106
: : . . . . . . *
YgbP A----ARPCLHQDDLARLLA-------LSETSRTGGI---LAAPVRDTMKRAEPGKNAIA 152
Cj1416 LIVSYADIVYFQDCVQKLINIKEELAIVVDKSWRKLWSKRFANPLEDAETLKMTNGYIIE 163
Oul-PntC LVVSESGIFSDLPSKSCYFG-------RMTEGYTDDWAF-----------DLDTTGRITR 142
Ari-PntC LVISDEHIFQRPRSRSCYFG-------RKFSGHTGDWVF-----------DLDDSGKIVR 158
Spn-LicC NYL-FKNMFRNDLTRSTYFS-------VYREDCTNEWFL-----------VYGDDYKVQD 143
Hin-LicC VVL-NRNIFLTKPSHSKYFT-------VIRSKTHNEWLP-----------ILNSNGQVIR 143
FrbH VVFEPAVLERLAAAPEASA--------VAASRPL--------RPLAGTVVRADADGVLTD 149
Bfr-PntC LVFERKAILSLLDDEFPDV--------MLYSSLT--------KFQDQYYVEYDRNHILTS 151
Tde_PntC LIYDSAGLFSLINDERKNL--------ILASGAT--------KSGDEVYLEADEKNCLTG 150
YgbP HTVDR----------NGLWHA-----LTPQFFPRELLH-DCLTRALNEGATITDEASAL- 195
Cj1416 LGKKANAYDEIEAQYIGLFK------FSYQFLSEVIAFYEMLDRDILY---DNKNFENM- 213
Oul-PntC VGKGAVNSYA----MVGISF------FKGGDAAHLARC---IRSAYTQ-----EGHERL- 183
Ari-PntC IGKGGSDTYA----MVGLSY------FSAPDAKRLARF---MHDAYKE-----TGHEQL- 199
Spn-LicC IIVDSKAGRI----LSGVSF------WDAPTAEKIVSF---IDKAY-----VSGEFVDL- 184
Hin-LicC IEIGSLNQSS----LSGVSY------WTTRDCDIILTL---LKEYTSE---VRLKNPKL- 186
FrbH YVDDRRQAG-AFDH-PGALKTANLYLLREAFLRERFLP------ALEE---LDRRLAGQG 198
Bfr-PntC CSVDKDALE-AKGELVGIHK------LSNTFYRRMCAD-------YAT---ILESQPKL- 193
Tde_PntC LSKNRDALKNIFGELVGITK------LTKSTLDKMCAY-------AKI---HHSDLPKM- 193
*
YgbP EYCGFHPQLVEGRADNIKVTRPEDLAL-----------AEFYLTRTIHQENT-- 236
Cj1416 YMTSFLQALIE--KYNNAKAVEIDGNWCEIDFMSDLEVQIDK------------ 253
Oul-PntC FWDDIVNQHID--EFDLRIRPVLEGQVVELDTIEELAAFDPSYSDQLRSNIDEG 235
Ari-PntC FWDDVVNNHIA--ELDLSIHPVEAQQIAELDSVAELAAFDHGYVYLLRS----- 246
Spn-LicC YWDNMVKDNIK--ELDVYVEELEGNSIYEIDSVQDYRKLEEILK----NEN--- 229
Hin-LicC YWDTIPMEYIE--KLNIYTEQLNSDDIFEMDNLDDYHHILQKLT----PNKEK- 233
FrbH YYDYAVSDGLAAGGHAWRVADISDLAWYEVDDPGDQRQAD-------------- 238
Bfr-PntC GYEYELLR-MSRSVSPVRVLRVEGLKWYEIDDEADLSYAEEHIIRYC------- 239
Tde_PntC EYEHALLE-AAK-TIPVAIKRIEYFVWREIDNEDHLEMA--------------- 230
AEP/ChoP binding
Metal coordination
Cytosine binding
Phosphate binding
Ribose binding
Apo salt bridge

B

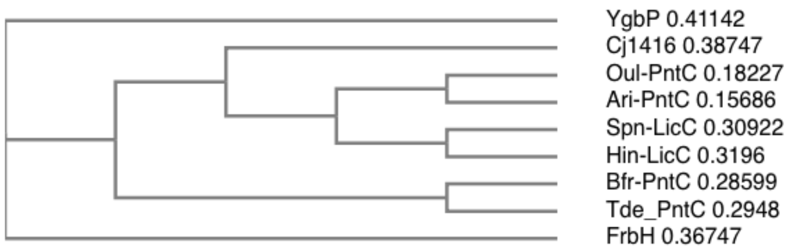

YgbP 0.41142
Cj1416 0.38747
Oul-PntC 0.18227
Ari-PntC 0.15686
Spn-LicC 0.30922
Hin-LicC 0.3196
Bfr-PntC 0.28599
Tde_PntC 0.2948
FrbH 0.36747

Supplementary Figure 15. (A) Clustal Omega multiple sequence alignment of selected PF12804 cytidylyltransferases. Amino acid sequences were retrieved from NCBI with the following accession numbers: Bfr-PntC from *Bacteroides fragilis* 638R (CBW22390); Tde-PntC from *Treponema denticola* ATCC 35405 (NP_992021); Cj1416 from *Campylobacter jejuni* (CAI38904); Oul-PntC from *Olsenella uli* DSM 7084 (ADK67708); Ari-PntC from *Atopobium rimae* ATCC 49626 (ZP_03568201); Spn-LicC from *Streptococcus pneumoniae* R36A (AAK94072); Hin-LicC from *Haemophilus influenzae* C486 (AJO89865); FrbH from *Streptomyces rubellomurinus* (ABB90397); YgbP (or IspD, CDP-ME synthase) from *Escherichia coli* K-12 (CQR82192). Box denotes the GXG(T/S)RX_8PK consensus sequence. The dotted line represents a salt bridge between Glu216 and Arg129 observed in the apo Spn-LicC crystal structure (PDB 1JYK).[3] (B) Cladogram of cytidylyltransferases from the alignment. YgbP is a MEP cytidylyltransferase belonging to the IspD Pfam (PF01128), which possesses significant overlap with PF12804.

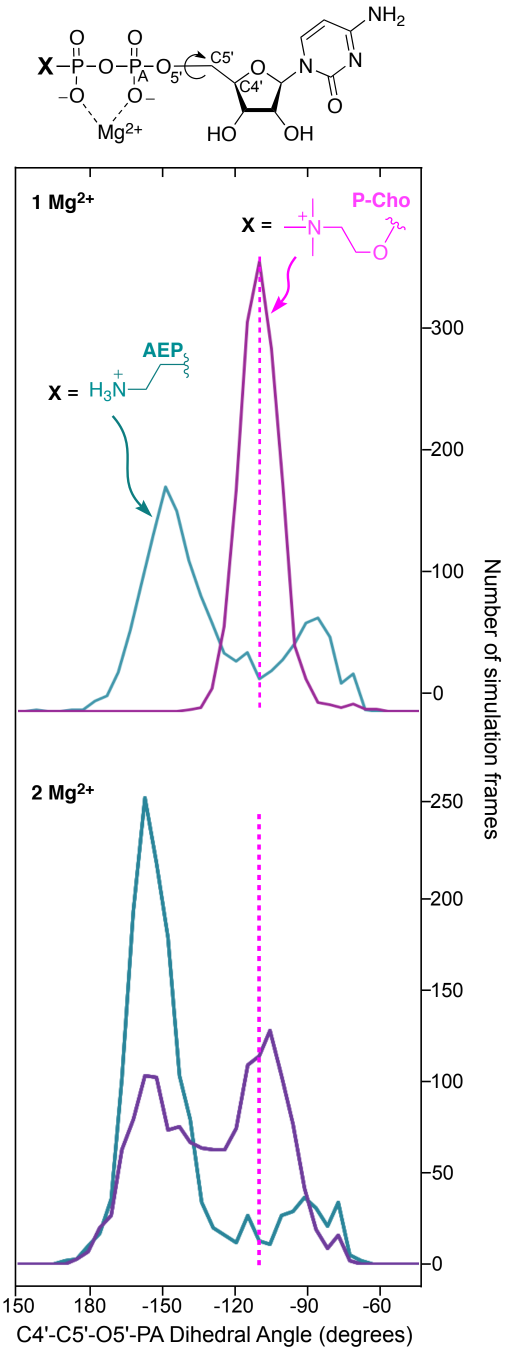

Supplementary Figure 16. The effects of a second Mg^{2+} ion on Spn-LicC MD simulations. The top panel reproduces the Spn-LicC data from Figure 5b in the main text, which resulted from simulations based on the crystallographic observation of a single Mg^{2+} ion in the active site. The bottom panel shows data resulting from the inclusion of a second Mg^{2+} ion in the simulations.

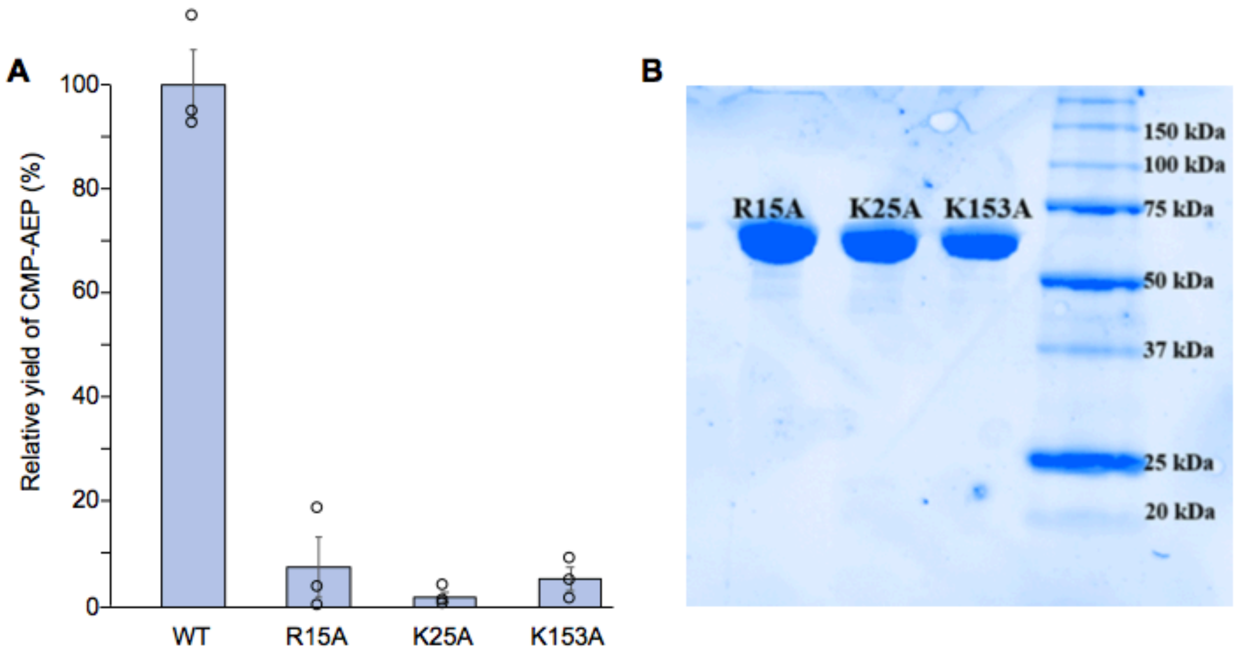

Supplementary Figure 17. Analysis of Tde1415 active site variants R15A, K25A, and K153A. (A) Activity of each variant based on integration of ^{31}P NMR peaks relative to a phosphonoacetic acid internal standard. Averages and standard errors of three measurements are shown as bars, with individual data points included as open circles. (B) SDS-PAGE analysis of purified variant enzymes.

Supplementary Table 1. X-ray data collection and refinement statistics (molecular replacement).*

	Tde-PntC-apo	Tde-PntC-CMP-AEP
**Data collection**		
Space group	$P2_1$	$P2_1$
Cell dimensions		
a, b, c (Å)	89.05, 129.01, 135.80	76.45, 154.05, 134.58
α, β, γ (°)	90, 92.99, 90	90, 90.09, 90
Resolution (Å)	38.00-2.72 (2.77-2.72)**	48.00-1.95 (2.06-1.95)
R_{merge}	0.097 (0.700)	0.188 (0.965)
$CC_{1/2}$	0.996 (0.728)	0.989 (0.767)
$I / \sigma I$	13.0 (2.1)	9.8 (2.1)
Completeness (%)	99.9 (99.7)	98.2 (97.1)
Redundancy	4.2 (4.3)	6.8 (7.0)
**Refinement**		
Resolution (Å)	2.72	1.95
No. reflections	342,389	1,510,620
R_{work} / R_{free}	0.199/0.233	0.220/0.254
No. atoms		
Protein (chains A, B, C, D)	19241	19277
Magnesium	4	8
PLP	-	64
CMP-AEP	-	108
gamma-PO_4	-	20
Water	257	1614
B-factors (Å^2)		
Protein (chains A, B, C, D)	48.7	26.4
Magnesium	55.9	28.9
PLP	-	22.1
CMP-AEP	-	31.4
gamma-PO_4	-	36.5
Water	48.6	28.7
R.m.s. deviations		
Bond lengths (Å)	0.005	0.011
Bond angles (°)	0.948	1.14

*One crystal was used for data collection and refinement. **Values in parentheses are for highest-resolution shell.

Supplementary Table 2. Strains used in this study.

Strain	Genotype/Description	Source (Reference)
E. coli DH5α	*E. coli* host for general cloning	Life Technologies
E. coli BL21(DE3)	*E. coli* host for protein production	Life Technologies
E. coli HG1000	pGH1000 in BL21(DE3)	This study
E. coli HG2000	pGH2000 in BL21(DE3)	This study
E. coli HG3000	pGH3000 in BL21(DE3)	This study
A. rimae ATCC 49626	VPI D140H-11A [NCFB 2896]	ATCC[4]
O. uli ATCC 49627	VPI D76D-27C^T = DSM 7084	ATCC[5]

Supplementary Table 3. Plasmids used in this study.

Plasmid	Description	Source (Reference)
pGH1000	pET29 plasmid containing *Spn-licC* as an *Nde*I/*Xho*I fragment	This study
pGH2000	pET29 plasmid containing *ari1348* as an *Nde*I/*Not*I fragment	This study
pGH3000	pET21 plasmid containing *tde1415* as an *Nde*I/*Not*I fragment	This study

Supplementary Table 4. Primers used in this study.

Target gene	Primer Name	Sequence (5'→3')
oul594	ouPEPmut-F	cgagaggatacggctctacg
	ouPEPmut-R	ctccatgatgtcgtctatcgtgc
oul592	ouPnPy decarb-F	atcacatcatcgccgcaaac
	ouPnPy decarb-R	tggtggagacgatggggtc
oul_rpoB	ourpoB-F	tcgacgtgcgcttcgtc
	ourpoB-R	gacacgcgcaaggtcg
oul602	ouLicC(602)-F	cgaggaggcgggtctg
	ouLicC(602)-R	gagtgatgcgaccgcc
oul609	ouLicD(609)-F	ctgcgtagggagggcg
	ouLicD(609)-R	gaaggtagggcatgcccg
oul593	ouLicC(593)-F	ccggtcaagctcatcgaaaac
	ouLicC(593)-R	ggacagggcggattctg
oul591	ouLicD(591)-F	ctgggatgatgatatcgacatcgg
	ouLicD(591)-R	tgtctgggtgaagcggttc
ari1347	arPEPmut-F	ctatctttacgacgacgtgattgc
	arPEPmut-R	tatccccgtcaagaatgatgggtttg
ari1349	arPnPy decarb-F	attatcaatcctgtggcttctcttctg
	arPnPy decarb-R	cgtcaccgtcaatgcacc
ari_rpoB	arrpoB-F	catgaccgagcgcgg
	arrpoB-R	gctatctcaagaccaagcttcttg
ari1348	arLicC-F	ggcgttgagaggatctctgtg
	arLicC-R	taattcggcaatatggttattcaccacatc
ari764	arLicD(764)-F	taccatctccatcggaatctttccg
	arLicD(764)-R	atctttccacttgccaaaatcaagtctg
ari768	arLicAC-F	ggactttcttctcgctttgctc
	arLicAC-R	cacggtcaaaatacacgtgtcc
ari769	arLicD(769)-F	cttgggacgatgatattgacgtcg
	arLicD(769)-R	tggtcttcacgtgtgcatagc
tde1415	Fwd-NdeI	catatgattaagcaagcggtgattctggc
	Rvs-XhoI	ctcgagaacgccaacgccgatgc
	Fwd-R15A	ctgggtagcgcactgaaggataagaccaagacc
	Rvs-R15A	ggtcttggtcttatccttcagtgcgctacccag
	Fwd-K25A	gaccaagaccatgccggcgggttttctggagatc
	Rvs-K25A	gatctccagaaaacccgccggcatggtcttggtc
	Fwd-K153A	ctgaccggcctgagcgcaaaccgtg
	Rvs-K153A	cacggtttgcgctcaggccggtcag

Supplementary References

1. Park, S.-N., Lim, Y. K. & Kook, J.-K. Development of quantitative real-time PCR primers for detecting 42 oral bacterial species. *Arch. Microbiol.* **195**, 473–482 (2013).
2. Weber, M. *et al.* Method development in quantitative NMR towards metrologically traceable organic certified reference materials used as (31)P qNMR standards. *Anal. Bioanal. Chem.* **407**, 3115–3123 (2015).
3. Kwak, B.-Y. *et al.* Structure and mechanism of CTP:phosphocholine cytidylyltransferase (LicC) from Streptococcus pneumoniae. *J. Biol. Chem.* **277**, 4343–4350 (2002).
4. Olsen, I., Johnson, J. L., Moore, L. V. & Moore, W. E. Lactobacillus uli sp. nov. and Lactobacillus rimae sp. nov. from the human gingival crevice and emended descriptions of lactobacillus minutus and Streptococcus parvulus. *Int. J. Syst. Bacteriol.* **41**, 261–266 (1991).
5. Göker, M. *et al.* Complete genome sequence of Olsenella uli type strain (VPI D76D-27C). *Stand. Genomic Sci.* **3**, 76–84 (2010).
