# Supplementary material for: The predominance of nucleotidyl activation in bacterial phosphonate biosynthesis
Source: Nat Commun. 2019 Aug 16;10:3698. doi: 10.1038/s41467-019-11627-6 (PMC6697681; doi:10.1038/s41467-019-11627-6)
Supplement: Supplementary file 2 — Reporting Summary [file 41467_2019_11627_MOESM2_ESM.pdf]

nature research

Corresponding author(s): Horsman, Geoff P.

Last updated by author(s): Jul 15, 2019

Reporting Summary

Nature Research wishes to improve the reproducibility of the work that we publish. This form provides structure for consistency and transparency in reporting. For further information on Nature Research policies, see Authors & Referees and the Editorial Policy Checklist.

Statistics

For all statistical analyses, confirm that the following items are present in the figure legend, table legend, main text, or Methods section.

n/a	Confirmed	
☐	☒	The exact sample size (*n*) for each experimental group/condition, given as a discrete number and unit of measurement
☒	☐	A statement on whether measurements were taken from distinct samples or whether the same sample was measured repeatedly
☒	☐	The statistical test(s) used AND whether they are one- or two-sided<br>*Only common tests should be described solely by name; describe more complex techniques in the Methods section.*
☒	☐	A description of all covariates tested
☒	☐	A description of any assumptions or corrections, such as tests of normality and adjustment for multiple comparisons
☐	☒	A full description of the statistical parameters including central tendency (e.g. means) or other basic estimates (e.g. regression coefficient) AND variation (e.g. standard deviation) or associated estimates of uncertainty (e.g. confidence intervals)
☒	☐	For null hypothesis testing, the test statistic (e.g. *F*, *t*, *r*) with confidence intervals, effect sizes, degrees of freedom and *P* value noted<br>*Give P values as exact values whenever suitable.*
☒	☐	For Bayesian analysis, information on the choice of priors and Markov chain Monte Carlo settings
☒	☐	For hierarchical and complex designs, identification of the appropriate level for tests and full reporting of outcomes
☒	☐	Estimates of effect sizes (e.g. Cohen's *d*, Pearson's *r*), indicating how they were calculated

Our web collection on statistics for biologists contains articles on many of the points above.

Software and code

Policy information about availability of computer code

Data collection	Enzyme kinetic data was collected using SoftMax Pro version XX. AutoProcess software was used to index and scale X-ray diffraction data.
Data analysis	HMMER version 3.1b2 software suite (includes HMMscan) was used to collect Hidden Markov Models from the Pfam database, retrieve homologs, and identify gene fusions. A custom Python script was written to filter results of HMMscan. A custom Perl script was written to account for overlapping domains in the fusion inventory. A custom Python script was written to calculate gene distances. Clustal Omega version 1.2.2 was used to construct multiple sequence alignment. The web-based POCASA program version 1.1 was used to calculate protein pocket volumes. PyMOL version 2.1.0 was used to visualize protein structures and build non-cogante ligands into enzyme active sites. The freely available Enlighten plugin for PyMOL (github.com/vanderkamp/enlighten2) was used to execute protocols of the AmberTools14 software. Crystal structures were solved by molecular replacement using the PHASER program version 2.7.0, and models were refined with PHENIX version 1.12 and COOT version 0.8.7.

For manuscripts utilizing custom algorithms or software that are central to the research but not yet described in published literature, software must be made available to editors/reviewers. We strongly encourage code deposition in a community repository (e.g. GitHub). See the Nature Research guidelines for submitting code & software for further information.

Data

Policy information about availability of data

All manuscripts must include a data availability statement. This statement should provide the following information, where applicable:

- Accession codes, unique identifiers, or web links for publicly available datasets
- A list of figures that have associated raw data
- A description of any restrictions on data availability

The authors declare that the data supporting the findings of this study are available within the paper and its supplementary information files of in a public repository. Specifically, associated raw data has been provided as a source data Excel file for Supplementary Figures 9, 14, and 17; data supporting the crystal structures of Tde1415 and Tde1415:CMP-AEP have been deposited in the protein data bank with respective PDB accession codes 6PD1 and 6PD2.

Field-specific reporting

Please select the one below that is the best fit for your research. If you are not sure, read the appropriate sections before making your selection.

☒ Life sciences ☐ Behavioural & social sciences ☐ Ecological, evolutionary & environmental sciences

For a reference copy of the document with all sections, see nature.com/documents/nr-reporting-summary-flat.pdf

Life sciences study design

All studies must disclose on these points even when the disclosure is negative.

Sample size	We always performed at least three replicate measurements.
Data exclusions	No data was excluded.
Replication	We always performed at least three replicate measurements.
Randomization	We did not work with live organisms so we did not use randomized trials.
Blinding	Blinding was not relevant because we did not use study organisms.

Reporting for specific materials, systems and methods

We require information from authors about some types of materials, experimental systems and methods used in many studies. Here, indicate whether each material, system or method listed is relevant to your study. If you are not sure if a list item applies to your research, read the appropriate section before selecting a response.

Materials & experimental systems

n/a	Involved in the study	
☒	☐	Antibodies
☒	☐	Eukaryotic cell lines
☒	☐	Palaeontology
☒	☐	Animals and other organisms
☒	☐	Human research participants
☒	☐	Clinical data

Methods

n/a	Involved in the study	
☒	☐	ChIP-seq
☒	☐	Flow cytometry
☒	☐	MRI-based neuroimaging
